# Supplementary material for: Computational prediction of high-risk non-synonymous SNPs in human ApoE and their structural impact on amyloid-β interaction in Alzheimer’s disease pathogenesis
Source: PLoS One. 2025 Sep 2;20(9):e0331339. doi: 10.1371/journal.pone.0331339 (PMC12404388; doi:10.1371/journal.pone.0331339)
Supplement: S1 File — S1 Table. Common deleterious nsSNPs identified by eight in-silico tools.S2 Table. Structural effect of 10 common deleterious nsSNPs over ApoE protein using Missense3D tool. S3 Table. MutPred2 analysis of 10 common deleterious nsSNPs identified in ApoE. S4 Table. Structural impact of 10 common deleterious nsSNPs on ApoE protein features predicted by HOPE. S5 Table. Active site residue, Surface area and Volume of WT ApoE, ApoE (L122P) and ApoE (L107P). S1 Fig: Validation of WT and mutant ApoE model by Ramachandran Plot. a) WT ApoE, b) ApoE (L122P) and c) ApoE (L107P). (DOCX) [file pone.0331339.s001.docx]

**S1 Table**. Common deleterious nsSNPs identified by eight *in-silico* tools.

| **SNP ID** | **AA Change** | **Position** | **SIFT** | **Polyphen-2** | **PredictSNP** | **PhD-SNP** | **PANTHER** | **SNAP2** | **Meta-SNP** | **PROVEAN** |
| --- | --- | --- | --- | --- | --- | --- | --- | --- | --- | --- |
| rs7412 | R/C | 202 | 0.66 | 0.003 | Deleterious | Neutral | Damaging | Effect (45) | 0 | -1.756 |
| rs429358 | C/R | 156 | 0.07 | 0.081 | Neutral | Neutral | Neutral | Neutral | 0 | -1.29 |
| rs440446 | N/K | 14 | 0.08 | 0 | Neutral | Neutral | Neutral | Neutral | 0.01 | -0.663 |
| rs769452 | L/Q | 72 | 0.01 | 0.335 | Neutral | Neutral | Neutral | Neutral | 0 | -0.71 |
| rs769455 | R/C | 189 | 0.09 | 0.881 | Deleterious | Neutral | Damaging | Effect (1) | 0.09 | -0.083 |
| rs11083750 | P/Q | 128 | 0.65 | 0.009 | Deleterious | Disease | Neutral | Neutral | 0.21 | 0.147 |
| rs11542029 | R/S | 76 | 0.84 | 1 | Deleterious | Neutral | Neutral | Effect (56) | 0.15 | -0.092 |
| rs11542041 | R/S | 158 | 0.6 | 0.005 | Deleterious | Neutral | Damaging | Effect (2) | 0.04 | -0.101 |
| rs28931576 | T/A | 86 | 0.04 | 0.081 | Neutral | Neutral | Neutral | Neutral | 0.01 | 0.002 |
| rs28931577 | A/T | 143 | 0.02 | 0 | Neutral | Neutral | Neutral | Neutral | 0.45 | -0.611 |
| rs28931578 | R/Q | 178 | 0.79 | 0.945 | Deleterious | Neutral | Neutral | Effect (1) | 0.1 | -0.686 |
| rs28931579 | S/R | 340 | 0.01 | 0.335 | Neutral | Neutral | Neutral | Neutral | 0.01 | -0.737 |
| rs121918392 | E/K | 47 | 0.13 | 0.823 | Neutral | Neutral | Damaging | Effect (56) | 0.46 | -0.433 |
| rs121918393 | R/S | 180 | 0.58 | 1 | Deleterious | Disease | Damaging | Effect (11) | 0.66 | -0.361 |
| rs121918394 | K/Q | 190 | 0.09 | 0.006 | Deleterious | Neutral | Neutral | Effect (45) | 0.65 | -0.068 |
| rs121918395 | R/S | 272 | 0.98 | 0.945 | Deleterious | Neutral | Neutral | Effect (8) | 0.02 | -0.388 |
| rs121918397 | R/H | 189 | 0.04 | 0.958 | Neutral | Neutral | Damaging | Neutral | 0.22 | 0.231 |
| rs121918398 | R/H | 318 | 0.66 | 0.966 | Deleterious | Neutral | Neutral | Effect (1) | 0.61 | -1.058 |
| rs121918399 | R/C | 69 | 0.6 | 0.057 | Deleterious | Neutral | Damaging | Effect (50) | 0.01 | -0.487 |
| rs140808909 | E/K | 288 | 0.04 | 0.958 | Deleterious | Neutral | Neutral | Effect (14) | 0.38 | -0.697 |
| rs190853081 | E/K | 289 | 0.01 | 0.653 | Deleterious | Neutral | Neutral | Effect (56) | 0.15 | -0.213 |
| rs199768005 | V/E | 280 | 0.58 | 0.966 | Deleterious | Neutral | Neutral | Effect (11) | 0.51 | -0.878 |
| rs200703101 | R/H | 180 | 0.02 | 0.897 | Deleterious | Disease | Damaging | Effect (45) | 0.65 | -7.058 |
| rs201672011 | E/K | 57 | 0.03 | 0.945 | Deleterious | Neutral | Neutral | Effect (14) | 0.01 | -0.046 |
| rs267606661 | R/G | 295 | 0.6 | 0.423 | Deleterious | Neutral | Damaging | Effect (78) | 0.85 | -1.72 |
| rs267606662 | A/P | 196 | 0.04 | 0.897 | Deleterious | Neutral | Damaging | Effect (1) | 0.17 | -0.694 |
| rs267606663 | R/Q | 268 | 0.01 | 0.003 | Neutral | Neutral | Damaging | Neutral | 0.45 | -0.509 |
| rs267606664 | G/D | 171 | 0.2 | 0.897 | Deleterious | Neutral | Neutral | Effect (40) | 0.18 | 0.496 |
| rs387906567 | R/G | 186 | 0.04 | 0.881 | Deleterious | Neutral | Damaging | Effect (45) | 0.64 | -1.591 |
| rs567353589 | E/K | 256 | 0.5 | 0.253 | Neutral | Neutral | Neutral | Neutral | 0.11 | 0.416 |
| rs587778876 | L/M | 148 | 0.58 | 0.958 | Deleterious | Neutral | Neutral | Effect (1) | 0.31 | -1.8 |
| rs587778877 | L/M | 177 | 0.66 | 0.653 | Deleterious | Neutral | Damaging | Neutral | 0 | -1.379 |
| rs745552623 | S/Y | 138 | 0 | 0.945 | Deleterious | Neutral | Damaging | Neutral | 0 | -1.576 |
| rs752790054 | R/H | 82 | 0.09 | 0.966 | Neutral | Neutral | Neutral | Neutral | 0.2 | 0.363 |
| rs767980905 | D/E | 109 | 0.06 | 0.003 | Neutral | Neutral | Neutral | Effect (45) | 1 | -1.975 |
| rs771354248 | E/D | 275 | 0.07 | 0 | Neutral | Neutral | Neutral | Neutral | 0.19 | -1.269 |
| rs1051068315 | S/R | 340 | 0.38 | 0.823 | Neutral | Neutral | Neutral | Neutral | 0.33 | -1.252 |
| rs1332591068 | R/P | 191 | 0.52 | 0.415 | Deleterious | Neutral | Neutral | Effect (56) | 0.3 | -0.838 |
| rs1969863273 | Q/R | 167 | 0.01 | 0.081 | Neutral | Neutral | Neutral | Neutral | 0 | -1.136 |
| rs2122137937 | G/A | 209 | 0.04 | 0.335 | Neutral | Neutral | Damaging | Neutral | 0.05 | -0.471 |
| rs11542027 | S/F | 241 | 0.79 | 0.003 | Deleterious | Neutral | Damaging | Neutral | 0.12 | 0.204 |
| rs11542030 | Q/R | 231 | 0.11 | 0.011 | Neutral | Neutral | Neutral | Neutral | 0.54 | -0.31 |
| rs11542032 | E/K | 215 | 0 | 0.958 | Deleterious | Neutral | Damaging | Effect (8) | 0.33 | -1.894 |
| rs11542034 | E/G | 176 | 0.84 | 0.653 | Deleterious | Neutral | Damaging | Effect (1) | 0.27 | -0.219 |
| rs11542035 | R/H | 163 | 0.04 | 0.999 | Neutral | Neutral | Neutral | Neutral | 0.01 | -2.192 |
| rs11542037 | R/G | 147 | 0.54 | 0.945 | Deleterious | Neutral | Damaging | Effect (45) | 0.07 | -1.377 |
| rs11542040 | P/T | 128 | 0.08 | 0.081 | Deleterious | Disease | Neutral | Neutral | 0.25 | -0.642 |
| rs41382345 | E/V | 165 | 0.66 | 0.823 | Deleterious | Disease | Damaging | Effect (56) | 0.27 | -0.113 |
| rs77903069 | D/E | 315 | 0.02 | 0.958 | Deleterious | Neutral | Neutral | Neutral | 0.42 | -0.118 |
| rs142480126 | E/K | 63 | 0.1 | 0.823 | Neutral | Neutral | Neutral | Neutral | 0.53 | -1.106 |
| rs144354013 | T/A | 37 | 0.03 | 0.897 | Neutral | Neutral | Neutral | Neutral | 1 | -0.401 |
| rs368210726 | L/S | 115 | 0.6 | 0.671 | Deleterious | Neutral | Damaging | Effect (45) | 0.37 | -2.474 |
| rs370594287 | Q/H | 90 | 0.05 | 0.823 | Deleterious | Neutral | Neutral | Neutral | 0.03 | -0.545 |
| rs371331933 | K/N | 119 | 0.58 | 0.653 | Deleterious | Neutral | Neutral | Effect (1) | 0.58 | -1.575 |
| rs371694216 | R/H | 69 | 0.4 | 0.966 | Neutral | Neutral | Neutral | Neutral | 0.19 | -1.567 |
| rs372938213 | A/E | 143 | 0.06 | 0.958 | Deleterious | Neutral | Damaging | Neutral | 0.03 | -1.542 |
| rs373985746 | G/E | 4 | 0.08 | 0 | Neutral | Neutral | Neutral | Effect (78) | 0 | 0.144 |
| rs376170967 | R/H | 194 | 0.04 | 0.966 | Deleterious | Neutral | Damaging | Neutral | 1 | -1.219 |
| rs387906568 | R/G | 268 | 0.66 | 0.881 | Deleterious | Neutral | Neutral | Effect (23) | 0.21 | -1.251 |
| rs530010303 | R/W | 261 | 0.79 | 0.999 | Deleterious | Neutral | Damaging | Effect (1) | 0.02 | -1.849 |
| rs531939919 | R/W | 178 | 0.01 | 0.644 | Deleterious | Disease | Damaging | Effect (12) | 0.68 | -8.349 |
| rs533904656 | A/T | 44 | 0 | 0.715 | Neutral | Neutral | Neutral | Neutral | 0.05 | -1.083 |
| rs539470710 | A/G | 330 | 0.05 | 0.653 | Neutral | Neutral | Neutral | Neutral | 0.06 | -1.192 |
| rs543363163 | G/S | 164 | 0.03 | 0.081 | Neutral | Neutral | Neutral | Neutral | 0.06 | -2.104 |
| rs547472686 | A/T | 251 | 0.79 | 0.003 | Deleterious | Neutral | Neutral | Effect (45) | 0.01 | -0.132 |
| rs551256627 | A/G | 300 | 0.01 | 0.958 | Neutral | Neutral | Neutral | Neutral | 0 | -1.616 |
| rs554251788 | R/G | 259 | 0.01 | 0.897 | Deleterious | Neutral | Neutral | Effect (11) | 0 | -2.378 |
| rs557715042 | W/C | 320 | 0 | 0.335 | Deleterious | Neutral | Neutral | Effect (1) | 0 | -1.635 |
| rs557845700 | M/I | 108 | 0.04 | 0.108 | Neutral | Neutral | Neutral | Neutral | 0.02 | -0.0988 |
| rs559532612 | A/T | 40 | 0.01 | 0.081 | Neutral | Neutral | Neutral | Neutral | 0.05 | -0.298 |
| rs563571689 | D/N | 19 | 0.8 | 0.003 | Neutral | Neutral | Neutral | Neutral | 0.02 | -2.057 |
| rs573658040 | R/G | 163 | 0.39 | 0.966 | Deleterious | Neutral | Neutral | Effect (45) | 0 | -1.507 |
| rs577618688 | Q/R | 125 | 0.03 | 0.945 | Deleterious | Neutral | Neutral | Effect (56) | 0.02 | 0.205 |
| rs745950059 | Q/R | 293 | 0.01 | 0.823 | Deleterious | Neutral | Neutral | Neutral | 0.25 | -2.086 |
| rs747078681 | F/Y | 38 | 0.08 | 0.958 | Neutral | Neutral | Neutral | Neutral | 0.04 | -2.136 |
| rs747975000 | V/I | 100 | 0.01 | 0.644 | Neutral | Neutral | Neutral | Neutral | 0.05 | -0.589 |
| rs748506927 | S/R | 219 | 0.44 | 0.975 | Deleterious | Neutral | Neutral | Neutral | 0.6 | -0.776 |
| rs748703149 | G/S | 171 | 0 | 0.897 | Neutral | Neutral | Neutral | Neutral | 0.4 | -0.723 |
| rs749102800 | V/M | 331 | 0.85 | 0.966 | Neutral | Neutral | Neutral | Neutral | 0.32 | -2.2 |
| rs749160976 | R/Q | 134 | 0.01 | 0 | Neutral | Neutral | Neutral | Neutral | 0.1 | -0.305 |
| rs749406635 | G/S | 67 | 0.52 | 0.081 | Neutral | Neutral | Neutral | Neutral | 0.58 | -1.916 |
| rs749750245 | R/S | 233 | 0.03 | 0.653 | Neutral | Neutral | Neutral | Effect (56) | 0.52 | -1.666 |
| rs750138933 | L/M | 312 | 0.03 | 0.275 | Neutral | Neutral | Neutral | Neutral | 0.06 | -0.269 |
| rs751200677 | Y/H | 206 | 0 | 0.966 | Deleterious | Neutral | Neutral | Effect (50) | 0.09 | -1.956 |
| rs752079771 | R/C | 59 | 0 | 0.999 | Deleterious | Neutral | Neutral | Neutral | 0.02 | -1.211 |
| rs752600356 | V/M | 155 | 0.82 | 0.644 | Deleterious | Neutral | Neutral | Effect (1) | 0 | -0.687 |
| rs753798476 | Q/R | 161 | 0.08 | 0.958 | Deleterious | Neutral | Neutral | Effect (45) | 0.23 | -1.673 |
| rs754211171 | A/T | 321 | 0.25 | 0.335 | Neutral | Neutral | Neutral | Neutral | 0.26 | -0.004 |
| rs754318486 | A/V | 33 | 0.71 | 0.06 | Neutral | Neutral | Neutral | Neutral | 0 | -2.424 |
| rs754627330 | Q/H | 207 | 0.03 | 0.881 | Neutral | Neutral | Neutral | Neutral | 0.03 | -0.781 |
| rs755792921 | V/M | 313 | 0.02 | 0.715 | Neutral | Neutral | Neutral | Neutral | 0 | -2.191 |
| rs755877232 | W/C | 20 | 0.06 | 0.081 | Deleterious | Disease | Neutral | Effect (56) | 0.02 | -2.247 |
| rs756353413 | Q/R | 65 | 0.06 | 0.056 | Neutral | Neutral | Neutral | Effect (45) | 0.13 | -1.804 |
| rs756564996 | R/H | 284 | 0.07 | 0.335 | Neutral | Neutral | Neutral | Neutral | 0.08 | -0.995 |
| rs757100480 | S/T | 98 | 0.05 | 0.011 | Neutral | Neutral | Neutral | Neutral | 0.16 | -1.499 |
| rs757764781 | G/R | 322 | 0.05 | 0.117 | Neutral | Neutral | Neutral | Neutral | 0.07 | -0.892 |
| rs757859088 | D/E | 198 | 0.01 | 0.335 | Neutral | Neutral | Neutral | Neutral | 0.79 | -1.745 |
| rs758487955 | K/R | 326 | 0.08 | 0.081 | Neutral | Neutral | Neutral | Neutral | 0.09 | -1.301 |
| rs759118026 | E/D | 310 | 0.08 | 0 | Neutral | Neutral | Neutral | Neutral | 0.06 | -0.734 |
| rs759501381 | P/A | 337 | 0.09 | 0.06 | Neutral | Neutral | Neutral | Neutral | 0.13 | -1.832 |
| rs759721023 | G/S | 240 | 0.09 | 0.003 | Neutral | Neutral | Neutral | Neutral | 0.11 | -0.91 |
| rs761285934 | K/T | 119 | 0.04 | 0.056 | Neutral | Neutral | Neutral | Neutral | 0.26 | -0.883 |
| rs761592007 | M/I | 265 | 0.09 | 0.335 | Neutral | Neutral | Neutral | Neutral | 0.15 | -1.552 |
| rs762461580 | R/H | 76 | 0.06 | 0.958 | Deleterious | Neutral | Neutral | Effect (1) | 0.15 | -0.798 |
| rs762703669 | Q/R | 102 | 0.03 | 0.653 | Neutral | Neutral | Neutral | Neutral | 0.03 | -1.32 |
| rs762845923 | R/C | 304 | 0.05 | 0.999 | Deleterious | Neutral | Damaging | Effect (56) | 0.07 | -0.337 |
| rs762906934 | E/K | 275 | 0.01 | 0.966 | Neutral | Neutral | Neutral | Neutral | 0.06 | -1.998 |
| rs762933906 | E/D | 175 | 0 | 0.975 | Neutral | Neutral | Neutral | Neutral | 0 | -1.172 |
| rs763313394 | D/N | 154 | 0.66 | 0.945 | Deleterious | Neutral | Damaging | Neutral | 0 | -2.269 |
| rs764929617 | P/L | 54 | 0.07 | 0.011 | Neutral | Neutral | Neutral | Neutral | 0.48 | -1.932 |
| rs765845034 | E/G | 275 | 0.05 | 0.081 | Deleterious | Neutral | Neutral | Effect (45) | 0.22 | -2.247 |
| rs767339630 | R/H | 304 | 0.09 | 0.644 | Deleterious | Neutral | Neutral | Effect (14) | 0.26 | -1.944 |
| rs767382895 | A/T | 130 | 0.04 | 0.958 | Neutral | Neutral | Neutral | Neutral | 0.05 | -2.304 |
| rs768780599 | L/Q | 95 | 0.06 | 0.715 | Deleterious | Neutral | Damaging | Effect (1) | 0.02 | -0.759 |
| rs768925016 | M/L | 169 | 0 | 0.653 | Neutral | Neutral | Neutral | Effect (40) | 0.07 | -1.154 |
| rs769366285 | V/A | 100 | 0.06 | 0.823 | Deleterious | Neutral | Damaging | Neutral | 0.06 | -1.012 |
| rs770485817 | R/C | 222 | 0.55 | 0.975 | Deleterious | Neutral | Damaging | Effect (56) | 0.13 | -0.361 |
| rs770545391 | E/K | 103 | 0.33 | 0.335 | Deleterious | Neutral | Damaging | Neutral | 0.13 | -0.627 |
| rs770562611 | T/I | 333 | 0 | 0.003 | Neutral | Neutral | Neutral | Neutral | 0.98 | 0.054 |
| rs770942678 | V/M | 234 | 0.01 | 0.644 | Neutral | Neutral | Neutral | Neutral | 0.84 | -0.895 |
| rs771594795 | K/E | 139 | 0.75 | 0.999 | Neutral | Neutral | Neutral | Neutral | 0.48 | -0.854 |
| rs772022603 | P/L | 18 | 0 | 0.06 | Neutral | Neutral | Neutral | Neutral | 0.46 | -1.437 |
| rs773797268 | E/G | 310 | 0.06 | 0.081 | Neutral | Neutral | Neutral | Neutral | 0.11 | -0.698 |
| rs774000134 | A/E | 106 | 0.71 | 0.335 | Neutral | Neutral | Neutral | Neutral | 0.01 | 0.086 |
| rs776242156 | A/V | 49 | 0.07 | 0.966 | Neutral | Neutral | Neutral | Neutral | 0.98 | -1.738 |
| rs777291619 | Q/P | 145 | 0.01 | 0.882 | Deleterious | Disease | Damaging | Effect (45) | 0.51 | -2.001 |
| rs778237451 | A/T | 285 | 0.44 | 0.958 | Neutral | Neutral | Neutral | Neutral | 0.2 | -0.25 |
| rs778348297 | R/Q | 136 | 0.05 | 0.653 | Neutral | Neutral | Neutral | Neutral | 0.28 | -0.629 |
| rs778425259 | R/H | 222 | 0.02 | 0.945 | Deleterious | Neutral | Damaging | Effect (13) | 0.61 | -0.36 |
| rs778901516 | W/C | 308 | 0.01 | 0.975 | Deleterious | Neutral | Damaging | Effect (45) | 0.57 | -1.493 |
| rs779278130 | L/P | 35 | 0.03 | 0.823 | Deleterious | Disease | Neutral | Effect (1) | 0.83 | -1.16 |
| rs779569800 | V/M | 166 | 0.07 | 0.958 | Neutral | Neutral | Neutral | Neutral | 0.13 | -0.458 |
| rs780035531 | E/K | 93 | 0 | 0.966 | Neutral | Neutral | Neutral | Neutral | 0.51 | -0.035 |
| rs780067631 | K/N | 326 | 0.04 | 0.081 | Deleterious | Neutral | Neutral | Effect (56) | 0.32 | -0.035 |
| rs780984110 | A/V | 281 | 0.05 | 0.003 | Neutral | Neutral | Neutral | Neutral | 0.32 | 0.859 |
| rs796443813 | A/P | 208 | 0.01 | 0.958 | Deleterious | Disease | Damaging | Effect (14) | 0.55 | -5.642 |
| rs867004984 | L/M | 185 | 0.03 | 0.999 | Neutral | Neutral | Neutral | Neutral | 0.55 | 0.282 |
| rs867215645 | K/N | 187 | 0.06 | 0.653 | Deleterious | Neutral | Damaging | Neutral | 0.61 | -0.193 |
| rs867594573 | R/S | 194 | 0.04 | 0.945 | Deleterious | Neutral | Damaging | Neutral | 0.37 | -1.207 |
| rs868094551 | H/N | 184 | 0.07 | 0.975 | Deleterious | Neutral | Damaging | Effect (1) | 0.13 | -0.18 |
| rs892532644 | E/D | 264 | 0.01 | 0.081 | Neutral | Neutral | Neutral | Neutral | 0.7 | -1.231 |
| rs916989869 | L/R | 225 | 0.08 | 0 | Deleterious | Disease | Damaging | Effect (40) | 0.33 | -0.484 |
| rs937011116 | R/C | 216 | 0.07 | 0.335 | Deleterious | Neutral | Damaging | Effect (56) | 0.16 | -1.312 |
| rs937063425 | A/V | 150 | 0 | 0.958 | Deleterious | Neutral | Neutral | Neutral | 0.18 | -0.605 |
| rs941248161 | Q/E | 245 | 0.07 | 0.999 | Neutral | Neutral | Neutral | Neutral | 0.25 | -0.894 |
| rs947015878 | A/G | 144 | 0.75 | 0.644 | Deleterious | Neutral | Damaging | Effect (45) | 0.27 | -0.208 |
| rs951182634 | E/G | 299 | 0.04 | 0.653 | Deleterious | Neutral | Neutral | Effect (78) | 0.27 | 0.486 |
| rs954186991 | A/T | 182 | 0.03 | 0.999 | Neutral | Neutral | Neutral | Neutral | 0.7 | -0.482 |
| rs954301448 | F/L | 309 | 0.06 | 0.966 | Deleterious | Neutral | Damaging | Effect (45) | 0.04 | -0.42 |
| rs981058595 | A/D | 210 | 0.05 | 0.975 | Deleterious | Neutral | Damaging | Effect (1) | 0.24 | -0.136 |
| rs988698841 | D/N | 198 | 0.06 | 0.999 | Deleterious | Neutral | Neutral | Neutral | 0.42 | 0.613 |
| rs990344075 | V/M | 179 | 0 | 0.958 | Neutral | Neutral | Neutral | Neutral | 0.54 | 0.547 |
| rs993409614 | D/N | 151 | 0.82 | 0.653 | Deleterious | Neutral | Damaging | Effect (56) | 0.4 | -0.147 |
| rs998239069 | S/T | 183 | 0.05 | 0.823 | Neutral | Neutral | Neutral | Neutral | 0.8 | -0.778 |
| rs1003367097 | R/C | 284 | 0.06 | 0.644 | Deleterious | Neutral | Damaging | Effect (45) | 0.08 | -1.893 |
| rs1009671345 | S/N | 267 | 0.08 | 0.011 | Neutral | Neutral | Neutral | Neutral | 0.01 | -1.423 |
| rs1018669382 | L/V | 181 | 0.01 | 0.335 | Neutral | Neutral | Neutral | Neutral | 0.17 | -0.571 |
| rs1023086190 | E/K | 110 | 0.02 | 0.081 | Neutral | Neutral | Neutral | Neutral | 0.13 | -1.13 |
| rs1032082950 | T/I | 174 | 0.99 | 0.882 | Neutral | Neutral | Neutral | Neutral | 0.01 | -2.33 |
| rs1039600156 | L/P | 323 | 0.03 | 0.958 | Deleterious | Disease | Damaging | Effect (23) | 0.58 | -3.435 |
| rs1039633588 | Q/P | 248 | 0.01 | 0.975 | Deleterious | Neutral | Neutral | Effect (11) | 0.16 | -2.084 |
| rs1047319304 | R/Q | 235 | 0.09 | 0.653 | Deleterious | Neutral | Damaging | Neutral | 0 | -1.434 |
| rs1048982607 | R/L | 224 | 0.53 | 0.823 | Deleterious | Neutral | Damaging | Effect (1) | 0.07 | -0.696 |
| rs1050106163 | R/W | 134 | 0.04 | 0.966 | Deleterious | Neutral | Damaging | Effect (45) | 0.01 | 0.527 |
| rs1052163943 | E/K | 94 | 0.06 | 0.081 | Neutral | Neutral | Neutral | Neutral | 0.46 | -1.637 |
| rs1162012100 | A/T | 168 | 0.05 | 0.056 | Neutral | Neutral | Neutral | Neutral | 0.09 | -0.519 |
| rs1164213063 | G/R | 226 | 0.01 | 0.06 | Neutral | Neutral | Neutral | Neutral | 0 | -0.87 |
| rs1164895411 | Q/R | 99 | 0.04 | 0.335 | Neutral | Neutral | Damaging | Neutral | 0.18 | -1.642 |
| rs1167428194 | K/T | 139 | 0.75 | 0.945 | Deleterious | Neutral | Damaging | Effect (50) | 0.01 | -2.103 |
| rs1169728519 | E/K | 140 | 0.08 | 0.011 | Deleterious | Neutral | Damaging | Effect (1) | 0.12 | -0.649 |
| rs1180612218 | Q/K | 125 | 0 | 0.003 | Neutral | Neutral | Neutral | Neutral | 0.29 | -0.691 |
| rs1181840153 | Q/K | 248 | 0.01 | 0 | Neutral | Neutral | Neutral | Neutral | 0.18 | -1.746 |
| rs1183932967 | E/V | 114 | 0.08 | 0.823 | Deleterious | Neutral | Neutral | Effect (56) | 0 | -2.43 |
| rs1184253344 | E/V | 94 | 0.88 | 0.958 | Deleterious | Neutral | Neutral | Effect (23) | 0.1 | -1.224 |
| rs1184454715 | I/M | 221 | 0.08 | 0.872 | Deleterious | Neutral | Neutral | Effect (45) | 0.23 | -0.592 |
| rs1190120890 | Q/K | 200 | 0.04 | 0.081 | Neutral | Neutral | Neutral | Neutral | 0 | -0.695 |
| rs1197351070 | E/V | 282 | 0.05 | 0.644 | Neutral | Neutral | Damaging | Neutral | 0.16 | -0.906 |
| rs1199910165 | A/V | 303 | 0.03 | 0.246 | Neutral | Neutral | Neutral | Neutral | 0.03 | -1.288 |
| rs1212454788 | R/H | 59 | 0.03 | 0.335 | Neutral | Neutral | Neutral | Neutral | 0.31 | -1.412 |
| rs1214824838 | G/V | 24 | 0.08 | 0.06 | Deleterious | Neutral | Neutral | Effect (56) | 0 | -0.953 |
| rs1217176327 | A/T | 298 | 0.01 | 0.011 | Neutral | Neutral | Neutral | Neutral | 0.07 | -1.228 |
| rs1221046029 | D/E | 274 | 0.41 | 0.013 | Neutral | Neutral | Neutral | Neutral | 0.04 | -2.479 |
| rs1227709957 | V/G | 239 | 0.03 | 0.999 | Deleterious | Neutral | Damaging | Effect (45) | 0.22 | -2.022 |
| rs1233347077 | S/N | 9 | 0.08 | 0.056 | Neutral | Neutral | Neutral | Neutral | 0.18 | -5.647 |
| rs1237339516 | R/Q | 257 | 0.08 | 0.335 | Neutral | Neutral | Neutral | Neutral | 0.03 | -6.655 |
| rs1237443001 | S/R | 267 | 0.02 | 0.945 | Neutral | Neutral | Neutral | Neutral | 0 | -3.707 |
| rs1238105907 | L/P | 122 | 0.01 | 1 | Deleterious | Disease | Damaging | Effect (45) | 0.78 | -5.775 |
| rs1238565583 | R/Q | 261 | 0.07 | 0.975 | Neutral | Neutral | Neutral | Neutral | 0.02 | -0.971 |
| rs1241692890 | A/T | 117 | 0.08 | 0.081 | Neutral | Neutral | Neutral | Neutral | 0.02 | -4.72 |
| rs1241924076 | A/T | 260 | 0.81 | 0.958 | Neutral | Neutral | Neutral | Neutral | 0 | -7.769 |
| rs1245215443 | E/G | 288 | 0.02 | 0.897 | Deleterious | Neutral | Damaging | Effect (1) | 0.01 | -5.761 |
| rs1245628517 | G/D | 332 | 0.05 | 0.011 | Neutral | Neutral | Neutral | Neutral | 0.22 | -8.585 |
| rs1246120787 | G/D | 164 | 0.01 | 0.975 | Neutral | Neutral | Neutral | Neutral | 0 | -5.72 |
| rs1246551383 | R/K | 7 | 0.03 | 0.335 | Neutral | Neutral | Neutral | Effect (56) | 0.01 | -4.691 |
| rs1249355043 | D/N | 197 | 0 | 0.644 | Deleterious | Neutral | Neutral | Neutral | 0.01 | 0.913 |
| rs1249808975 | Q/R | 61 | 0.05 | 0.823 | Neutral | Neutral | Neutral | Neutral | 1 | -1.992 |
| rs1263042140 | R/H | 158 | 0.04 | 0.966 | Deleterious | Neutral | Damaging | Effect (45) | 0.09 | -2.818 |
| rs1265280650 | E/Q | 89 | 0.99 | 0.653 | Deleterious | Neutral | Neutral | Neutral | 0.15 | -1.804 |
| rs1265472491 | G/S | 217 | 0.05 | 0 | Neutral | Neutral | Neutral | Neutral | 0.02 | -3.754 |
| rs1265743589 | P/L | 246 | 0.01 | 0.715 | Neutral | Neutral | Neutral | Effect (1) | 0.01 | -1.854 |
| rs1271901056 | G/R | 149 | 0.08 | 0.003 | Neutral | Neutral | Neutral | Neutral | 0.01 | -2.606 |
| rs1275147925 | T/A | 23 | 0.02 | 0.081 | Neutral | Neutral | Neutral | Neutral | 0.27 | -3.756 |
| rs1276043726 | A/V | 260 | 0.08 | 0.958 | Deleterious | Neutral | Neutral | Neutral | 0 | -4.691 |
| rs1276509170 | A/T | 210 | 0.01 | 0.823 | Neutral | Neutral | Neutral | Neutral | 0.01 | -0.592 |
| rs1278944082 | K/E | 116 | 0.06 | 0.872 | Neutral | Neutral | Neutral | Neutral | 0.13 | -5.224 |
| rs1280930848 | S/R | 9 | 0.05 | 0.335 | Deleterious | Neutral | Neutral | Effect (45) | 0.02 | -3.747 |
| rs1282294107 | M/K | 316 | 0.07 | 0.966 | Deleterious | Neutral | Neutral | Effect (56) | 0 | -4.431 |
| rs1282920511 | M/I | 316 | 0.03 | 0.081 | Neutral | Neutral | Neutral | Neutral | 0.05 | -1.797 |
| rs1287096724 | L/I | 170 | 0 | 0.06 | Neutral | Neutral | Neutral | Neutral | 0.6 | -4.635 |
| rs1289377244 | L/R | 177 | 0.02 | 1 | Deleterious | Disease | Damaging | Effect (1) | 0.38 | -5.2 |
| rs1298395482 | A/S | 329 | 0.08 | 0.975 | Neutral | Neutral | Neutral | Neutral | 0.16 | -4.885 |
| rs1299844242 | G/R | 255 | 0.06 | 0.011 | Neutral | Neutral | Neutral | Neutral | 0.04 | -7.271 |
| rs1300558735 | Y/C | 118 | 0.06 | 0.823 | Deleterious | Disease | Damaging | Effect (56) | 0.03 | -4.663 |
| rs1300930146 | D/Y | 195 | 0.04 | 0.653 | Deleterious | Neutral | Damaging | Effect (11) | 0.04 | -4.161 |
| rs1301223903 | P/S | 17 | 0.01 | 0.335 | Neutral | Neutral | Neutral | Neutral | 0.02 | -3.872 |
| rs1301411037 | W/L | 64 | 0.05 | 0.003 | Neutral | Neutral | Neutral | Effect (45) | 0.12 | -2.192 |
| rs1303558981 | E/G | 223 | 0.02 | 0.081 | Neutral | Neutral | Neutral | Effect (40) | 0.08 | -5.713 |
| rs1307832357 | R/H | 259 | 0.01 | 0.06 | Neutral | Neutral | Neutral | Neutral | 0 | -7.604 |
| rs1324343215 | A/S | 146 | 0.05 | 0.999 | Neutral | Neutral | Neutral | Neutral | 0.06 | -6.666 |
| rs1325189389 | P/T | 311 | 0 | 0.945 | Deleterious | Disease | Neutral | Neutral | 0 | -5.713 |
| rs1327865483 | V/L | 129 | 0.02 | 0.644 | Neutral | Neutral | Neutral | Neutral | 0 | -4.747 |
| rs1330045687 | A/V | 135 | 0.06 | 0.823 | Deleterious | Neutral | Damaging | Neutral | 0 | -3.389 |
| rs1330553726 | R/H | 272 | 0.03 | 0.081 | Neutral | Neutral | Neutral | Neutral | 0.01 | -5.739 |
| rs1332509626 | G/A | 213 | 0.09 | 0.011 | Neutral | Neutral | Neutral | Neutral | 0 | -5.553 |
| rs1334498236 | D/N | 271 | 0.06 | 0.335 | Neutral | Neutral | Neutral | Neutral | 0.06 | -5.553 |
| rs1334600571 | E/K | 114 | 0 | 0.958 | Deleterious | Neutral | Neutral | Effect (50) | 0.06 | -3.684 |
| rs1335550286 | E/K | 263 | 0.07 | 0.653 | Deleterious | Neutral | Neutral | Effect (1) | 0 | -2.307 |
| rs1336089822 | P/S | 12 | 0.01 | 0 | Neutral | Neutral | Neutral | Effect (78) | 0.01 | -3.609 |
| rs1339651557 | R/H | 211 | 0.01 | 0.081 | Neutral | Neutral | Damaging | Neutral | 0 | -5.38 |
| rs1340326154 | V/G | 331 | 0.03 | 0.999 | Deleterious | Neutral | Neutral | Effect (56) | 0 | -1.8 |
| rs1341982092 | Y/C | 162 | 0.04 | 0.823 | Deleterious | Disease | Damaging | Effect (14) | 0.65 | -5.779 |
| rs1350058178 | Q/E | 292 | 0.03 | 0.945 | Neutral | Neutral | Neutral | Neutral | 0 | -6.128 |
| rs1351828960 | A/T | 335 | 0.05 | 0.958 | Neutral | Neutral | Neutral | Neutral | 0.05 | -0.666 |
| rs1355806321 | R/C | 318 | 0.08 | 0.999 | Deleterious | Neutral | Damaging | Effect (23) | 0.05 | -0.323 |
| rs1356186009 | G/D | 149 | 0.08 | 0.966 | Neutral | Neutral | Neutral | Neutral | 0.18 | -1.71 |
| rs1357260388 | G/V | 255 | 0.08 | 0.003 | Deleterious | Neutral | Neutral | Neutral | 0.44 | -3.31 |
| rs1358158446 | P/H | 311 | 0.02 | 0.653 | Deleterious | Disease | Damaging | Effect (56) | 0.51 | -4.89 |
| rs1360151558 | A/S | 253 | 0.01 | 0.975 | Neutral | Neutral | Neutral | Neutral | 0.5 | -3.339 |
| rs1367811482 | E/Q | 325 | 0.06 | 0.335 | Neutral | Neutral | Neutral | Neutral | 0.77 | -2.501 |
| rs1367830766 | C/Y | 156 | 0.04 | 0.958 | Deleterious | Neutral | Neutral | Neutral | 0.82 | -4.861 |
| rs1369662219 | E/Q | 132 | 0.09 | 0.823 | Deleterious | Neutral | Damaging | Effect (1) | 0.04 | -2.757 |
| rs1376916580 | A/T | 237 | 0.05 | 0.966 | Neutral | Neutral | Neutral | Neutral | 0.02 | -7.049 |
| rs1377502673 | S/F | 6 | 0.08 | 0.081 | Neutral | Neutral | Neutral | Effect (12) | 0.04 | -3.672 |
| rs1377553244 | K/N | 116 | 0.01 | 0.335 | Neutral | Neutral | Neutral | Neutral | 0.01 | -5.509 |
| rs1377830202 | L/M | 188 | 0.07 | 0.653 | Neutral | Neutral | Neutral | Neutral | 0 | -6.876 |
| rs1378119160 | V/M | 327 | 0.06 | 0.318 | Neutral | Neutral | Neutral | Neutral | 0 | -7.228 |
| rs1380773651 | I/F | 221 | 0.55 | 0.966 | Neutral | Neutral | Neutral | Neutral | 0.23 | -4.508 |
| rs1381171305 | R/W | 257 | 0 | 0.975 | Deleterious | Neutral | Damaging | Effect (56) | 0.66 | -1.727 |
| rs1381224336 | F/L | 38 | 0.55 | 0.003 | Neutral | Neutral | Neutral | Neutral | 0.75 | -2.877 |
| rs1385100920 | G/D | 232 | 0 | 0.653 | Deleterious | Neutral | Damaging | Effect (8) | 0.85 | -1.836 |
| rs1388704077 | G/R | 209 | 0.01 | 0.975 | Deleterious | Neutral | Damaging | Effect (1) | 0 | -2.185 |
| rs1391215146 | G/D | 266 | 0.03 | 0.966 | Deleterious | Neutral | Neutral | Effect (78) | 0.55 | -7.221 |
| rs1394832846 | V/M | 50 | 0.56 | 0.897 | Neutral | Neutral | Neutral | Neutral | 0 | -5.167 |
| rs1396111554 | E/K | 310 | 0.04 | 0.945 | Deleterious | Neutral | Neutral | Effect (40) | 0.92 | -4.352 |
| rs1402219759 | R/W | 191 | 0.02 | 0.958 | Deleterious | Neutral | Damaging | Effect (56) | 0.01 | -0.704 |
| rs1402325936 | E/V | 215 | 0.09 | 0.653 | Deleterious | Neutral | Damaging | Effect (50) | 0.58 | -4.261 |
| rs1407867184 | M/R | 108 | 0.04 | 0.823 | Deleterious | Neutral | Damaging | Effect (1) | 0.88 | -5.501 |
| rs1413700881 | T/N | 269 | 0 | 0.197 | Deleterious | Neutral | Neutral | Effect (56) | 0.74 | -2.05 |
| rs1418355128 | E/D | 94 | 0.57 | 0 | Neutral | Neutral | Neutral | Neutral | 0.88 | -2.807 |
| rs1418673754 | V/G | 237 | 0.02 | 0.966 | Deleterious | Neutral | Damaging | Effect (48) | 0 | -5.661 |
| rs1421977676 | V/A | 205 | 0.06 | 0.715 | Neutral | Neutral | Neutral | Neutral | 0.01 | -2.095 |
| rs1424027593 | L/M | 141 | 0.03 | 0.653 | Neutral | Neutral | Neutral | Effect (23) | 0.81 | -1.948 |
| rs1425703838 | R/P | 250 | 0.06 | 0.999 | Deleterious | Neutral | Damaging | Effect (11) | 0.58 | 1.147 |
| rs1426176730 | L/F | 96 | 0.04 | 0.245 | Neutral | Neutral | Neutral | Neutral | 0.26 | -2.579 |
| rs1426426514 | R/C | 224 | 0.08 | 0.644 | Deleterious | Neutral | Damaging | Effect (1) | 0.82 | -1.676 |
| rs1429543001 | A/D | 146 | 0.01 | 0.081 | Neutral | Neutral | Neutral | Neutral | 0.06 | -1.777 |
| rs1434244093 | A/T | 5 | 0.01 | 0.011 | Neutral | Neutral | Neutral | Neutral | 0.85 | -1.777 |
| rs1434287349 | M/V | 112 | 0.58 | 0.823 | Deleterious | Neutral | Damaging | Effect (56) | 1 | -6.847 |
| rs1434405741 | Q/K | 207 | 0.05 | 0.335 | Neutral | Neutral | Neutral | Neutral | 0.01 | -4.246 |
| rs1438607869 | E/K | 175 | 0.04 | 0.999 | Deleterious | Neutral | Damaging | Effect (11) | 0.74 | -3.124 |
| rs1439276876 | L/V | 74 | 0.03 | 0.003 | Neutral | Neutral | Neutral | Neutral | 0.04 | -1.301 |
| rs1440369210 | G/R | 266 | 0.04 | 0.958 | Neutral | Neutral | Neutral | Effect (56) | 0.64 | -1.33 |
| rs1440786605 | E/K | 124 | 0.02 | 0.081 | Neutral | Neutral | Neutral | Neutral | 0 | -0.613 |
| rs1440976751 | E/K | 51 | 0.01 | 0.011 | Neutral | Neutral | Neutral | Neutral | 0.71 | -0.641 |
| rs1448845160 | A/V | 130 | 0.06 | 0.945 | Neutral | Neutral | Neutral | Neutral | 0.03 | -0.859 |
| rs1452005331 | R/H | 186 | 0.02 | 0.975 | Deleterious | Neutral | Damaging | Neutral | 0.22 | -1.06 |
| rs1453797593 | E/D | 278 | 0.59 | 0.06 | Neutral | Neutral | Neutral | Neutral | 0.3 | 1.156 |
| rs1456562720 | Q/H | 48 | 0.07 | 0.999 | Neutral | Neutral | Neutral | Neutral | 0.66 | -4.785 |
| rs1457217956 | L/P | 185 | 0.04 | 0.958 | Deleterious | Disease | Damaging | Effect (1) | 0.72 | -5.828 |
| rs1458301734 | G/S | 157 | 0.08 | 0.081 | Neutral | Neutral | Neutral | Neutral | 0.14 | -6.647 |
| rs1459489355 | R/H | 233 | 0.06 | 0.653 | Neutral | Neutral | Neutral | Effect (23) | 0.07 | -7.68 |
| rs1459595735 | R/W | 235 | 0.06 | 0.958 | Deleterious | Neutral | Damaging | Effect (56) | 1 | -3.124 |
| rs1461741495 | V/I | 36 | 0.02 | 0.011 | Neutral | Neutral | Neutral | Neutral | 0.77 | -7.573 |
| rs1463421531 | S/R | 307 | 0.06 | 0.823 | Deleterious | Neutral | Neutral | Effect (1) | 0.06 | -3.345 |
| rs1463848099 | A/T | 291 | 0.03 | 0.066 | Neutral | Neutral | Neutral | Neutral | 0.88 | -0.899 |
| rs1466115957 | G/D | 244 | 0 | 0.644 | Deleterious | Neutral | Neutral | Effect (12) | 0.01 | -0.881 |
| rs1466963971 | L/Q | 141 | 0.04 | 0.975 | Deleterious | Neutral | Damaging | Effect (45) | 0.1 | -1.628 |
| rs1468448662 | T/K | 23 | 0.01 | 0 | Neutral | Neutral | Neutral | Effect (11) | 0.99 | -1.06 |
| rs1470816009 | S/I | 2 | 0.07 | 0.003 | Neutral | Neutral | Neutral | Effect (56) | 0.2 | -1.246 |
| rs1471997722 | L/P | 247 | 0.02 | 0.653 | Deleterious | Disease | Damaging | Effect (23) | 0.73 | -0.613 |
| rs1476757984 | L/F | 305 | 0 | 0.999 | Neutral | Neutral | Neutral | Neutral | 0.07 | -1.509 |
| rs1479320041 | S/N | 307 | 0.04 | 0.958 | Neutral | Neutral | Neutral | Neutral | 0.52 | -0.354 |
| rs1483485796 | S/C | 183 | 0.05 | 0.945 | Deleterious | Neutral | Damaging | Neutral | 0.6 | -4.24 |
| rs1486760107 | V/L | 338 | 0.65 | 0.823 | Neutral | Neutral | Neutral | Neutral | 0.6 | -2.392 |
| rs1488379910 | G/S | 244 | 0.03 | 0.081 | Neutral | Neutral | Neutral | Neutral | 0.05 | 0.071 |
| rs1555790637 | M/I | 262 | 0.06 | 0.897 | Neutral | Neutral | Neutral | Neutral | 0.26 | -0.927 |
| rs1568615382 | A/T | 32 | 0.01 | 0.011 | Neutral | Neutral | Neutral | Neutral | 0.71 | -1.987 |
| rs1568615443 | L/R | 39 | 0.66 | 0.958 | Deleterious | Disease | Damaging | Effect (45) | 0.07 | -2.667 |
| rs1568618190 | D/A | 315 | 0.02 | 0.056 | Deleterious | Disease | Damaging | Effect (56) | 0.01 | -0.613 |
| rs1599950832 | V/G | 36 | 0.06 | 0.644 | Deleterious | Disease | Damaging | Effect (1) | 0.01 | -0.847 |
| rs1599951908 | G/D | 67 | 0.03 | 0.081 | Neutral | Neutral | Neutral | Neutral | 0.55 | -1.756 |
| rs1599951966 | E/G | 71 | 0.02 | 0 | Deleterious | Disease | Damaging | Effect (45) | 0 | -1.29 |
| rs1599952017 | G/S | 75 | 0.01 | 0.335 | Neutral | Neutral | Neutral | Neutral | 0.01 | -0.663 |
| rs1599952025 | G/D | 75 | 0.05 | 0.715 | Neutral | Neutral | Neutral | Neutral | 0 | -0.71 |
| rs1599952129 | E/D | 89 | 0.01 | 0.011 | Neutral | Neutral | Neutral | Neutral | 0.09 | -0.083 |
| rs1599952206 | L/H | 96 | 0.06 | 0.823 | Neutral | Neutral | Neutral | Neutral | 0.21 | 0.147 |
| rs1599952234 | Q/K | 99 | 0.08 | 0.999 | Neutral | Neutral | Damaging | Neutral | 0.15 | -0.092 |
| rs1599952958 | T/I | 127 | 0.05 | 0.653 | Neutral | Neutral | Neutral | Neutral | 0.04 | -0.101 |
| rs1599953056 | A/T | 135 | 0.04 | 0.958 | Neutral | Neutral | Neutral | Neutral | 0.01 | 0.002 |
| rs1599953294 | G/D | 157 | 0.67 | 0.003 | Neutral | Neutral | Neutral | Neutral | 0.45 | -0.611 |
| rs1599953509 | V/A | 179 | 0.08 | 0.081 | Neutral | Neutral | Neutral | Neutral | 0.1 | -0.686 |
| rs1599953786 | D/E | 197 | 0.03 | 0.335 | Neutral | Neutral | Neutral | Neutral | 0.01 | -0.737 |
| rs1599953984 | L/V | 218 | 0.03 | 0.823 | Neutral | Neutral | Neutral | Neutral | 0.66 | -0.433 |
| rs1599954003 | A/T | 220 | 0.01 | 0.862 | Neutral | Neutral | Neutral | Neutral | 0.66 | -0.361 |
| rs1599954391 | V/G | 283 | 0.06 | 0.945 | Deleterious | Neutral | Damaging | Effect (56) | 0.65 | -0.068 |
| rs1599954500 | A/T | 300 | 0.03 | 0.644 | Neutral | Neutral | Neutral | Neutral | 0.02 | -0.388 |
| rs1599954557 | S/G | 307 | 0.68 | 0.011 | Neutral | Neutral | Neutral | Neutral | 0.22 | 0.231 |
| rs1599954663 | Q/R | 317 | 0.05 | 0.081 | Neutral | Neutral | Neutral | Effect (45) | 0.61 | -1.058 |
| rs1599954673 | Q/H | 317 | 0.01 | 0.06 | Neutral | Neutral | Neutral | Neutral | 0.01 | -0.487 |
| rs1599954804 | S/R | 334 | 0.09 | 0.958 | Neutral | Neutral | Neutral | Neutral | 0.8 | -0.697 |
| rs1969797567 | A/D | 5 | 0.03 | 0.011 | Neutral | Neutral | Neutral | Effect (1) | 0.15 | -0.213 |
| rs1969797737 | S/A | 6 | 0.01 | 0.056 | Neutral | Neutral | Neutral | Neutral | 0.51 | -0.878 |
| rs1969798111 | S/R | 9 | 0.05 | 0.999 | Deleterious | Neutral | Damaging | Effect (48) | 0.66 | -1.058 |
| rs1969798162 | W/R | 10 | 0.08 | 0.653 | Deleterious | Neutral | Damaging | Effect (56) | 0.91 | -0.046 |
| rs1969798360 | G/R | 13 | 0.06 | 0.823 | Deleterious | Neutral | Damaging | Effect (12) | 0.85 | -1.72 |
| rs1969798659 | P/L | 17 | 0.06 | 0.011 | Neutral | Neutral | Neutral | Neutral | 0.17 | -0.694 |
| rs1969812567 | A/T | 33 | 0.01 | 0.081 | Neutral | Neutral | Neutral | Neutral | 0.55 | -0.509 |
| rs1969834815 | V/G | 50 | 0.04 | 0.999 | Deleterious | Neutral | Neutral | Neutral | 0.18 | 0.496 |
| rs1969835105 | T/A | 52 | 0.68 | 0.335 | Neutral | Neutral | Neutral | Neutral | 0.64 | -1.591 |
| rs1969835176 | P/S | 54 | 0.08 | 0.056 | Neutral | Neutral | Neutral | Neutral | 0.11 | 0.416 |
| rs1969836611 | Q/K | 68 | 0.06 | 0.644 | Deleterious | Neutral | Damaging | Effect (56) | 0.31 | -4.8 |
| rs1969838529 | D/V | 79 | 0.09 | 0.011 | Neutral | Neutral | Damaging | Neutral | 0.55 | -3.379 |
| rs1969838696 | R/C | 82 | 0.02 | 0.653 | Deleterious | Neutral | Damaging | Neutral | 0.66 | -2.176 |
| rs1969840702 | S/F | 98 | 0.05 | 0.958 | Deleterious | Neutral | Damaging | Effect (45) | 0.2 | 0.363 |
| rs1969841781 | R/K | 105 | 0.01 | 0 | Neutral | Neutral | Neutral | Neutral | 1 | -1.975 |
| rs1969853500 | A/S | 106 | 0.01 | 0.003 | Neutral | Neutral | Neutral | Neutral | 0.69 | -1.269 |
| rs1969853804 | L/P | 107 | 0.01 | 1 | Deleterious | Disease | Damaging | Effect (1) | 0.73 | -4.252 |
| rs1969854498 | M/I | 112 | 0.69 | 0.653 | Deleterious | Neutral | Damaging | Effect (56) | 0.83 | -1.838 |
| rs1969855667 | E/K | 121 | 0.04 | 0.966 | Deleterious | Neutral | Damaging | Effect (23) | 0 | -1.136 |
| rs1969855742 | E/D | 121 | 0.03 | 0.897 | Neutral | Neutral | Neutral | Neutral | 0.85 | -0.471 |
| rs1969858652 | E/G | 140 | 0.02 | 0.975 | Deleterious | Neutral | Damaging | Effect (48) | 0.72 | 0.204 |
| rs1969859756 | R/Q | 147 | 0.07 | 0.872 | Deleterious | Neutral | Damaging | Effect (14) | 0.54 | -0.31 |
| rs1969862344 | Y/H | 162 | 0.07 | 0.958 | Deleterious | Neutral | Neutral | Effect (56) | 0.63 | -1.894 |
| rs1969866587 | H/Q | 184 | 0.01 | 0.644 | Neutral | Neutral | Neutral | Neutral | 0.27 | -2.219 |
| rs1969867147 | K/R | 187 | 0.05 | 0.653 | Deleterious | Neutral | Neutral | Neutral | 0.51 | -2.192 |
| rs1969869057 | A/V | 196 | 0.01 | 0.715 | Neutral | Neutral | Neutral | Neutral | 0.07 | -1.377 |
| rs1969871875 | E/K | 212 | 0.06 | 0.966 | Deleterious | Neutral | Neutral | Effect (1) | 0.55 | -0.642 |
| rs1969872015 | G/R | 213 | 0.11 | 0.653 | Deleterious | Neutral | Neutral | Effect (45) | 0.77 | -0.113 |
| rs1969876660 | Q/H | 245 | 0.04 | 0.897 | Deleterious | Neutral | Damaging | Effect (23) | 0.72 | -0.118 |
| rs1969876805 | L/V | 247 | 0.06 | 0.975 | Neutral | Neutral | Neutral | Neutral | 0.53 | -1.106 |
| rs1969877509 | W/G | 254 | 0.09 | 0.823 | Deleterious | Disease | Neutral | Effect (56) | 1 | -0.401 |
| rs1969880357 | T/S | 269 | 0.04 | 0.653 | Neutral | Neutral | Neutral | Neutral | 0.7 | -2.474 |
| rs1969880574 | R/G | 270 | 0.04 | 0.966 | Deleterious | Neutral | Neutral | Effect (14) | 0.07 | -0.545 |
| rs1969881429 | V/A | 276 | 0 | 0.081 | Neutral | Neutral | Neutral | Neutral | 0.58 | -2.475 |
| rs1969881980 | V/M | 283 | 0.01 | 0.003 | Neutral | Neutral | Neutral | Neutral | 0.99 | -1.567 |
| rs1969883949 | Q/P | 297 | 0.05 | 0.644 | Deleterious | Neutral | Damaging | Effect (1) | 0.63 | -0.542 |
| rs1969884484 | F/L | 301 | 0.06 | 0.823 | Neutral | Neutral | Neutral | Neutral | 0.25 | 0.144 |
| rs1969885568 | W/G | 308 | 0.03 | 0.653 | Deleterious | Disease | Damaging | Effect (12) | 1 | -6.219 |
| rs1969888430 | Q/R | 328 | 0.03 | 0.975 | Deleterious | Neutral | Neutral | Neutral | 0.61 | -1.251 |
| rs1969889418 | A/T | 336 | 0.08 | 0.335 | Neutral | Neutral | Neutral | Neutral | 0.72 | -2.849 |
| rs1969889684 | P/L | 337 | 0.04 | 0.823 | Deleterious | Neutral | Neutral | Neutral | 0 | -1.349 |
| rs1969890013 | S/N | 340 | 0.06 | 0.644 | Neutral | Neutral | Neutral | Neutral | 0.35 | -1.083 |
| rs2122126151 | M/I | 1 | 0.01 | 0 | Deleterious | Neutral | Neutral | Neutral | 0.66 | -2.361 |
| rs2122126407 | N/S | 14 | 0.03 | 0.081 | Neutral | Neutral | Neutral | Neutral | 0.06 | -1.804 |
| rs2122132426 | Q/H | 60 | 0.06 | 0.975 | Deleterious | Neutral | Neutral | Effect (45) | 0.71 | -3.132 |
| rs2122132454 | T/A | 62 | 0.01 | 0.653 | Neutral | Neutral | Neutral | Neutral | 0 | -0.616 |
| rs2122135745 | T/P | 133 | 0.04 | 0.958 | Deleterious | Neutral | Damaging | Effect (1) | 0 | -2.378 |
| rs2122136031 | Q/P | 142 | 0.02 | 0.823 | Deleterious | Disease | Damaging | Effect (45) | 0 | -1.635 |
| rs2122137485 | L/P | 192 | 0 | 0 | Deleterious | Disease | Damaging | Effect (14) | 0.02 | -2.988 |
| rs2122137508 | L/P | 193 | 0.03 | 0.999 | Deleterious | Neutral | Neutral | Neutral | 0.05 | -1.298 |
| rs2122137873 | A/V | 208 | 0.09 | 0.945 | Neutral | Neutral | Neutral | Neutral | 0.02 | -2.057 |
| rs2122138074 | A/P | 214 | 0.04 | 0.003 | Deleterious | Disease | Damaging | Effect (12) | 0 | -1.507 |
| rs2122138444 | Q/E | 231 | 0.01 | 0 | Neutral | Neutral | Neutral | Neutral | 0.02 | 0.205 |
| rs2122138694 | S/P | 241 | 0.07 | 0.644 | Deleterious | Neutral | Neutral | Effect (1) | 0.25 | -2.086 |
| rs2122139524 | R/H | 270 | 0.08 | 0.715 | Deleterious | Disease | Neutral | Effect (56) | 0.74 | -1.136 |
| rs2122140552 | A/V | 321 | 0.05 | 0.872 | Deleterious | Neutral | Neutral | Neutral | 0.85 | -0.589 |

Highlighted SNP ID indicates selected nsSNP for further study

**S2 Table**. Structural effect of 10 common deleterious nsSNPs over ApoE protein using Missense3D tool.

| **SNP ID** | **AA Change** | **Chain** | **Results analysis** | **Detailed analysis** |
| --- | --- | --- | --- | --- |
| rs200703101 | R180H | A | No Structural damage detected. | **------** |
| rs531939919 | R178W | A | No Structural damage detected. | ------ |
| rs796443813 | A208P | A | Clash. | This substitution triggers clash alert. The local clash score for wild type is 54.60 and the local clash score for mutant is 74.74. |
| rs1039600156 | L323P | A | Cavity altered. | The substitution leads to the expansion of cavity volume by 74.088 Å^3. |
| rs1238105907 | L122P | A | Buried Pro introduced.  Secondary structure altered. | This substitution introduces a buried proline.  This substitution changes 'H' (4-turn helix) to 'T' (hydrogen bonded turn). |
| rs1341982092 | Y162C | A | No Structural damage detected. | ------ |
| rs1358158446 | P311H | A | Secondary structure altered. | This substitution changes 'T' (hydrogen bonded turn) to 'H' (4-turn helix). |
| rs1457217956 | L185P | A | No Structural damage detected. | ------ |
| rs1969853804 | L107P | A | Buried Pro introduced. | This substitution introduces a buried proline. |
| rs1969885568 | W308G | A | Cavity altered | The substitution leads to the expansion of cavity volume by 73.872 Å^3. |

**S3 Table**. MutPred2 analysis of 10 common deleterious nsSNPs identified in ApoE.

| **SNP ID** | **AA Change** | **(g-score)** | **Top Features with P-value** |
| --- | --- | --- | --- |
| rs200703101 | R180H | 0.117 | No Alteration |
| rs531939919 | R178W | 0.178 | No Alteration |
| rs796443813 | A208P | 0.219 | No Alteration |
| rs1039600156 | L323P | 0.578 | Altered Metal binding (P= 0.03) |
|  |  |  | Altered Stability (P= 0.04) |
| rs1238105907 | L122P | 0.797 | Loss of SUMOylation at K119 (P= 0.01) |
|  |  |  | Altered Coiled coil (P= 0.02) |
|  |  |  | Altered Stability (P= 0.02) |
|  |  |  | Altered Transmembrane protein (P= 0.02) |
|  |  |  | Gain of B-factor (P= 8.0e-03) |
|  |  |  | Altered Disordered interface (P= 0.04) |
| rs1341982092 | Y162C | 0.329 | No Alteration |
| rs1358158446 | P311H | 0.452 | No Alteration |
| rs1457217956 | L185P | 0.422 | No Alteration |
| rs1969853804 | L107P | 0. 596 | Altered Coiled coil (P= 7.0e-03) |
|  |  |  | Gain of Intrinsic disorder (P= 7.8e-03) |
|  |  |  | Altered Transmembrane protein (P= 8.3e-05) |
|  |  |  | Altered Stability (P= 4.8e-03) |
|  |  |  | Altered Disordered interface (P= 4.0e-03) |
| rs1969885568 | W308G | 0.622 | Altered Transmembrane protein (P= 7.8e-05) |
|  |  |  | Altered Metal binding (P= 0.02) |
|  |  |  | Altered Ordered interface (P= 0.02) |
|  |  |  | Altered DNA binding (P= 0.01) |

**S4 Table**. Structural impact of 10 common deleterious nsSNPs on ApoE protein features predicted by HOPE.

| **SNP ID** | **AA Change** | **Affects size and charge** | **Affect hydrophobicity** | **Disrupt H_2_ bond/Salt bridge** | **Interferes with protein function** | **Interferes with other protein interaction** |
| --- | --- | --- | --- | --- | --- | --- |
| rs200703101 | R180H | Only affects size | No | No | No | No |
| rs531939919 | A208P | Yes | No | No | Yes | Yes |
| rs796443813 | L323P | Yes | No | No |  | No |
| rs1039600156 | L122P | Yes | Yes | Disrupts H_2_ bond | Yes | Yes |
| rs1238105907 | R178W | Only affects size | No | No | No | No |
| rs1341982092 | Y162C | Only affects size | No | No | No | No |
| rs1358158446 | P311H | Only affects size | No | No | No | No |
| rs1457217956 | L185P | Yes | No | No | No | No |
| rs1969853804 | L107P | Yes | Yes | Disrupts H_2_ bond | Yes | Yes |
| rs1969885568 | W308G | Only affects size | No | No | No | No |

**S5 Table:** Active site residue, Surface area and Volume of WT ApoE, ApoE (L122P) and ApoE (L107P).

| **ApoE (L122P)** | | **WT ApoE** | | **ApoE (L107P)** | |
| --- | --- | --- | --- | --- | --- |
| **Pocket 1** | | **Pocket 1** | | **Pocket 1** | |
| **Area (SA) (Å^2^)** | **Volume (SA) (Å^3^)** | **Area (SA) (Å^2^)** | **Volume (SA) (Å^3^)** | **Area (SA) (Å^2^)** | **Volume (SA) (Å^3^)** |
| 101.561 | 47.649 | 70.701 | 30.859 | 70.996 | 31.422 |
| **Seq ID** | **Amino acid** | **Seq ID** | **Amino acid** | **Seq ID** | **Amino acid** |
| 115 | LEU | 119 | LYS | 119 | LYS |
| 118 | TYR | 119 | LYS | 119 | LYS |
| 118 | TYR | 119 | LYS | 119 | LYS |
| 119 | LYS | 122 | LEU | 122 | LEU |
| 119 | LYS | 122 | LEU | 122 | LEU |
| 119 | LYS | 122 | LEU | 122 | LEU |
| 119 | LYS | 123 | GLU | 123 | GLU |
| 119 | LYS | 123 | GLU | 123 | GLU |
| 119 | LYS | 123 | GLU | 123 | GLU |
| 122 | PRO | 126 | LEU | 126 | LEU |
| 122 | PRO | 126 | LEU | 126 | LEU |
| 123 | GLU | 134 | ARG | 134 | ARG |
| 123 | GLU | 134 | ARG | 134 | ARG |
| 123 | GLU | 134 | ARG | 134 | ARG |
| 126 | LEU | 134 | ARG | 134 | ARG |
| 126 | LEU | 134 | ARG | 134 | ARG |
| 134 | ARG | 137 | LEU | 137 | LEU |
| 134 | ARG | 137 | LEU | 137 | LEU |
| 134 | ARG | 138 | SER | 138 | SER |
| 134 | ARG | 138 | SER | 138 | SER |
| 134 | ARG | 138 | SER | 138 | SER |
| 137 | LEU | 141 | LEU | 141 | LEU |
| 137 | LEU |  |  |  |  |
| 138 | SER |  |  |  |  |
| 138 | SER |  |  |  |  |
| 138 | SER |  |  |  |  |
| 141 | LEU |  |  |  |  |
| 141 | LEU |  |  |  |  |
| 141 | LEU |  |  |  |  |
| 141 | LEU |  |  |  |  |
| 145 | GLN |  |  |  |  |
| 145 | GLN |  |  |  |  |


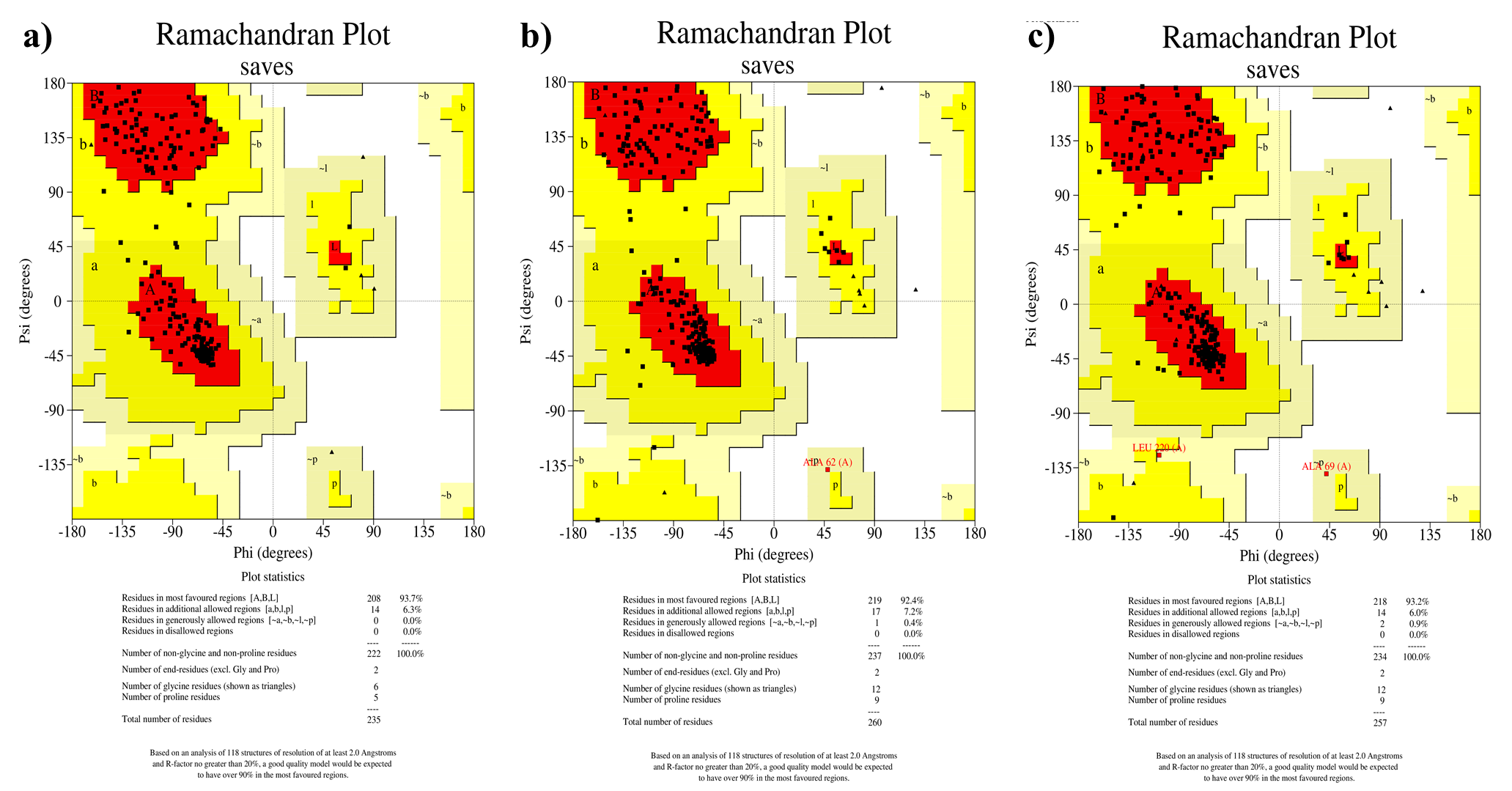


**S1 Fig:** Validation of WT and mutant ApoE model by Ramachandran Plot. a) WT ApoE, b) ApoE (L122P) and c) ApoE (L107P).
