# Supplementary material for: Computational prediction of high-risk non-synonymous SNPs in human ApoE and their structural impact on amyloid-β interaction in Alzheimer’s disease pathogenesis
Source: PLoS One. 2025 Sep 2;20(9):e0331339. doi: 10.1371/journal.pone.0331339 (PMC12404388; doi:10.1371/journal.pone.0331339)
Supplement: Attachment — Submitted filename: Response to Reviewers.docx [file pone.0331339.s002.docx]

21 July 2025

Manuscript Number: PONE-D-25-29477

Type of manuscript: Article

Title: Computational Prediction of High-risk Non-Synonymous SNPs in Human ApoE and Their Impact on Amyloid β binding, a causative agent of Alzheimer's disease

Dear Editor,

Thank you very much for reviewing our submission titled, “**Computational Prediction of High-risk Non-Synonymous SNPs in Human ApoE and Their Impact on Amyloid β binding, a causative agent of Alzheimer's disease**”. We thank all reviewers for their very constructive comments and suggestions and revised the manuscript accordingly (marked by track change function in Microsoft word in the revised manuscript). Each reviewer’s comments have been addressed below. We feel these changes have significantly strengthened this manuscript and hope that it will now be suitable for publication in biomedicines.

**Reviewer 01#**

**General comments:**

**Q1]** The uploaded figures are mainly low resolution and not vivid enough. Make sure to update the figures with the high-resolution version later on.

**Response**: Thank you for your valuable feedback regarding the quality of the figures. In response, we updated all figures with high-resolution.

**Specific Comments:**

**Material and Methods:**

**Q1]** There are quite significant numbers of different isoforms for ApoE. You can check it in here:
<https://www.ncbi.nlm.nih.gov/protein/?term=apolipoprotein+E+isoform>. What is your consideration and justification to pick NP_001289617.1?

**Response:** We thank the reviewer. We selected NP_001289617.1 for this study, as it encodes a full-length ApoE isoform containing all key functional domains involved in lipid metabolism, receptor binding, and amyloid-beta (Aβ) interaction. Compared to the shorter canonical isoform NP_000032.1, it includes additional N-terminal residues that may influence structural integrity and ligand binding. NP_001289617.1, derived from transcript NM_001302688.1, is well-characterized and closely linked to ApoE4-associated Alzheimer's disease (AD) pathology. Other isoforms (e.g., NP_001289618.1–NP_001289620.1), generated through alternative splicing, often lack essential regions or show limited relevance to AD (https://www.ensembl.org/). Furthermore, NP_001289617.1 demonstrates consistent brain expression and is frequently studied in the context of neurodegeneration. According to the reviewer’s suggestion, we included above section into our revised manuscript version **(page 6, line 181-191**).

**Q2]** Kindly mention that all the deployed programs were for benchmarking purposes.

**Response:** Thank you for your response. It should be noted that all the deployed programs in this study were utilized solely for benchmarking purposes and not intended for direct clinical or operational application (**page 7, line 211-213**).

**Q3]** Table 1: Only PredictSNP and SNAP2 got accuracy rates above 80%. Why not use only them for the computational work?

**Response:** Thank you for your constructive feedback. Although PredictSNP and SNAP2 demonstrated the highest accuracy rates (>80%) in this study (Table 1), relying solely on these tools could introduce bias and limit the diversity of computational assessments. By employing a combination of multiple predictive algorithms, we ensure a more comprehensive, cross-validated, and methodologically robust evaluation, reducing the likelihood of false predictions and enhancing confidence in the overall results.

Homology modelling, validation, and molecular docking study:

**Q4]** How did you validate your docking protocol? What standard and/or method do you use?

**Response:**

Our docking protocol employed AutoDock Vina v1.2.0, a widely validated and robust docking engine, within the PyRx platform. AutoDock Vina's accuracy in predicting ligand-receptor interactions and binding affinities is well-established through extensive testing against experimental datasets. Its gradient-descent optimization and sophisticated scoring function, approximating binding free energy, have demonstrated good correlation with experimental results, making it a de-facto standard.

Validation of our protocol relies on AutoDock Vina's inherent reliability and widespread acceptance, coupled with our comprehensive grid box definition (center: X: 18.1270, Y: 39.719, Z: 52.6633; dimensions: X: 53.4957, Y: 27.6560, Z: 48.9882 Angstrom). This grid ensured exhaustive exploration of potential binding sites across the entire ApoE protein, minimizing the omission of relevant binding poses. While experimental validation was outside the scope of this computational study, the use of a thoroughly validated software and comprehensive search strategy ensures the computational validity and reliability of our predictions. Ligand-receptor interactions were subsequently analyzed using Discovery Studio for qualitative assessment of binding mode plausibility.

**Q5]** How will you determine the binding of the A-beta peptide to the ApoE protein? How to validate the binding?

**Response**: Thank you for your question. According to your suggestion we performed molecular dynamic simulation for best protein complex to determine stability for 100 Nano-second that are included into our revised manuscript version (**page 12, line 332-347 and page 26, 27, 27 28, line 708-783**).

We determined the binding of the amyloid-beta (Aβ) peptide to ApoE proteins (WT, L107P, and L122P) through molecular docking analysis using PyRx virtual screening software combined with AutoDock Vina v1.2.0. This computational approach quantifies binding strength by calculating binding affinity (kcal/mol), where a more negative value indicates stronger binding. As presented in Table 5, the binding affinities were −5.5 kcal/mol for WT ApoE, −5.6 kcal/mol for ApoE L107P, and −6.6 kcal/mol for ApoE L122P.

Binding was further characterized by analyzing the specific molecular interactions between amyloid-beta (Aβ) and the active site residues of each ApoE variant. This included identifying hydrogen bonds, alkyl bonds, Pi-Sigma interactions, and Van der Waals forces, as illustrated in Fig 8. For instance, WT ApoE interacted with amyloid-beta (Aβ) via GLU 140 and ARG 147 (hydrogen bonds), while mutant ApoE L122P formed hydrogen bonds with TYR 118, GLN 125, and THR 127.

Validation of the binding is primarily based on the established reliability of AutoDock Vina. This software is widely recognized and extensively validated in computational biology for its accuracy in predicting ligand-receptor interactions and approximating binding free energies. Our protocol also involved a comprehensive grid box search to ensure all potential binding sites were explored, contributing to the robustness of our predictions. The observed correlation between the active site changes (CASTpFold analysis, Fig 7) induced by the L122P mutation and the significantly enhanced binding affinity further supports the validity of our findings. While experimental validation (e.g., ITC, SPR) was beyond the scope of this computational study, our methodology adheres to accepted computational standards for predicting and characterizing protein-peptide interactions.

Result:

**Q6]** Table 3: What can you infer from this benchmarking table? Which one is the best in terms of time and/or space complexity? Which one is the most suitable for providing biological insight? Kindly narrate accordingly!

**Response:** Thank you for your question. Most tools consistently predict destabilizing effects for SNPs like (L122P), L323P, L107P, and W308G. MutPred2 ranks L122P highest in pathogenic potential (g-score 0.797) and variants introducing proline (e.g., L122P, L107P) significantly alter stability and secondary structure. Overall, rs1238105907 (L122P) and L107P are the most damaging, and combining results from multiple tools ensures robust inference.

MutPred2 is accurate but slower due to its large feature set. Structure-based tools like DynaMut2, Missense3D and HOPE are more accurate but computationally intensive. On the other hand, tools like I-MUTANT and MUpro are computationally efficient, offering faster predictions with lower space complexity.

MutPred2 is the most suitable tool for biological insight as it predicts the functional impact of nsSNPs beyond just stability. It provides a g-score indicating potential pathogenicity and suggests molecular mechanisms affected. Unlike tools like I-Mutant or MUpro, which focus on ΔΔG values, MutPred2 links mutations to biological consequences. It helps prioritize disease-relevant variants. Thus, it offers the most comprehensive functional interpretation.

**Q7]** Kindly mention the CASTpFOLD tool-related pipeline in the methodology!

**Response:** Thank you for your valuable suggestion. The CASTpFold tool-related pipeline has now been incorporated into the methodology section into the revised methodology (**page 11, line 308-311**).

“To identify active and binding sites, we utilized the CASTpFold server (https://cfold.bme.uic.edu/castpfold/), which integrates protein folding and cavity topography information to accurately map pockets, cavities, and channels in protein structures. CASTpFold was applied to both WT and the mutant ApoE variants (L122P and L107P)”.

**Reviewer 02#**

Abstract:

**Q1]** The abstract provides a comprehensive summary, but it can be made more concise. For example, replace “forecast their potential impact” with “predict their impact.”.

**Response:** Thank you for your constructive feedback. We have revised the phrase “forecast their potential impact” to “predict their impact” in our revised manuscript (**page 1, line 28**).

**Q2]** Revise the aim statement for precision:

Suggested: "This study aimed to identify functionally damaging nsSNPs in the ApoE gene using in silico tools and to assess their structural and binding effects on amyloid-beta (Aβ) in the context of Alzheimer’s disease progression".

**Response:** Thank you for your insightful suggestion regarding the aim statement.

"This study aimed to identify functionally damaging nsSNPs in the ApoE gene using in silico tools and to assess their structural and binding effects on Aβ in the context of AD progression."

We incorporated above statement into our abstract section (**page 1, line 27-29**).

**Q3]** Emphasize the significance of the receptor-binding domain by briefly noting its importance in mediating ApoE-Aβ interaction.

**Response:** Thank you for your valuable suggestion. In response, we incorporated the following statement into the abstract of the revised manuscript (**page 2, line 42 and 43**).

“The receptor-binding domain mediates the interaction between ApoE and Aβ.”

**Q4]** Explain the biological implication of the L122P mutation (e.g., stronger binding affinity may promote Aβ aggregation or hinder clearance, potentially contributing to disease severity).

**Response:** Thank you for the insightful comment. According to your suggestion, we included the biological implication of the L122P mutation into the abstract of the revised manuscript (**page 2, line 44-46**).

“Stronger binding affinity of L122P mutation may promote Aβ aggregation or hinder clearance, potentially contributing to disease severity.”

**Introduction**

**Q1]** Add a brief comparison with previously characterized pathogenic ApoE variants (e.g., C112R, R158C) to provide context and highlight novelty.

**Response:** Thank you for your thoughtful suggestion. According to your suggestion, we added a brief comparison with previously characterized pathogenic ApoE variants (e.g., C112R, R158C) to provide context and highlight novelty into our revised manuscript (**page 4, line 125-128**).

“previously studied variants such as C112R (rs429358) and R158C (rs7412) have been shown to shift ApoE’s domain interactions and affect its lipidation status and aggregation propensity, contributing to AD pathophysiology.”

**Q2]** Elaborate on how missense mutations in ApoE disrupt receptor binding or lipid transport, which are essential for Aβ metabolism and clearance.

**Response:** We totally agree with your valuable suggestion. We elaborated this statement into our revised manuscript version (**page 4, line 119-122**)

“Missense mutations in ApoE can perturb receptor binding, lipid transport, and Aβ interactions, functions that are particularly mediated by its receptor-binding domain (residues 1-167), a critical region for interaction with low-density lipoprotein receptors and Aβ peptides.”

**Q3]** Improve flow by separating the general background from the study objective:

Suggested: "Given the limitations of experimental approaches, this study employed in silico tools to identify deleterious nsSNPs in ApoE and evaluate their potential structural and functional effects on Aβ interaction."

**Response:** Thank you for your helpful suggestion. We separated the general background from the study objective into our revised manuscript (**page 5, line 151-153**).

“Given the limitations of experimental approaches, this study employed in silico tools to identify deleterious nsSNPs in ApoE and evaluate their potential structural and functional effects on Aβ interaction.”

**Q4]** Eliminate redundant phrasing related to Aβ accumulation and its role in AD (lines 2–7). For example, “Aβ peptide buildup and aggregation in the extracellular region” and “progressive buildup and aggregation in the brain” convey similar meanings and can be merged more concisely.

**Response:** Thank you for your careful observation. We eliminated redundant phrasing related to Aβ accumulation and its role in AD (**page 3, line 70-82**)

**Q5]** Add the chromosomal location of ApoE (19q13.32) and include key SNPs (rs429358 and rs7412) that define ApoE2, E3, and E4 isoforms.

Response: Thank you for your valuable suggestion. We added the chromosomal location of ApoE (19q13.32) and include key SNPs (rs429358 and rs7412) that define ApoE2, E3, and E4 isoforms into our revised manuscript (**page 3, line 91-96**).

“The ApoE gene is located on chromosome 19q13.32 and encodes the apolipoprotein E protein, a 299-amino acid glycoprotein involved in lipid metabolism, synaptic maintenance, and clearance of Aβ peptides from the brain. The three major ApoE isoforms ApoE2, ApoE3, and ApoE4 are defined by two single nucleotide polymorphisms (SNPs): rs429358 (C112R) and rs7412 (R158C), which produce distinct amino acid substitutions at positions 112 and 158.”

**Q6]** Improve the transition from general SNP discussion to ApoE-specific focus.

Suggested: "Among genes linked to neurodegeneration, ApoE stands out due to its central role in Alzheimer’s disease, especially through structural changes introduced by specific nsSNPs."

**Response:** Thank you for your thoughtful suggestion. We incorporated this suggestion into our revised manuscript version (**page 4, line 111-113**).

“Among genes linked to neurodegeneration, ApoE stands out due to its central role in AD, especially through structural changes introduced by specific non-synonymous single nucleotide polymorphisms (nsSNPs).”

**Methodology**

**Q1]** Clearly state whether population-level association data (e.g., allele frequency or GWAS statistics) are included. If not, mention this limitation.

**Response:** Thank you for highlighting this important point. We clearly stated that this study focuses primarily on in silico functional and structural analyses of nsSNPs in the ApoE gene; however, population-level association data, including allele frequencies and genome-wide association study (GWAS) statistics, were not incorporated and represent a limitation of the current work (**page 6, line 177-180**).

**Q2]** Describe the criteria for identifying and selecting nsSNPs (e.g., ClinVar significance, allele frequency thresholds).

**Response:** Thank you for your insightful comment. We described the criteria identifying and selecting nsSNPs into the revised manuscript version.

Criteria for Identifying and Selecting nsSNPs

1. Database Screening: Select nsSNPs from databases like dbSNP, ClinVar, and DisGeNET focusing on coding regions that result in amino acid changes.
2. Functional Impact Prediction: Use tools like SIFT, PolyPhen-2, or PROVEAN to identify variants predicted to be deleterious or damaging.
3. Protein Stability and Structure: Assess changes in protein stability (e.g., via I-Mutant, MUpro) and focus on SNPs in functional domains.

**Q3]** Explain why each computational tool was chosen, emphasizing their complementarity (sequence-based vs. structure-based) and reliability.

**Response:** Thank you for your valuable comment. We have now clarified the rationale for selecting each computational tool, highlighting their complementary strengths and reliability into our revised manuscript methodology section (**page 7, line 208-214**).

“By integrating multiple computational algorithms (sequence-based vs. structure-based), the reliability of variant classification is substantially enhanced. Consistent predictions across diverse tools strengthen the evidence supporting the pathogenicity of a given variant, while discordant results may reveal distinct mechanistic interpretations, such as protein destabilization versus impaired receptor binding. This integrative, multi-tool strategy provides a more comprehensive and refined assessment of the functional implications of genetic variants”.

**Q4]** Consider incorporating DynaMut to analyse mutation-induced changes in protein flexibility and entropy.

**Response:** Thank you for your valuable recommendation.

We used DynaMut2 analysis for analyse mutation-induced changes in protein flexibility and entropy and subsequently, the results added into our revised manuscript (**page 9, line240, 249-251**).

**Q5]** Include URLs for all tools used to improve reproducibility (e.g., SIFT: <http://sift.jcvi.org/>).

**Response:** Thank you for your valuable suggestion. We included URLs for all tools used improve reproducibility into our revised manuscript version.

dbSNP: <https://www.ncbi.nlm.nih.gov/snp/>

ClinVar: <https://www.ncbi.nlm.nih.gov/clinvar/>

DisGeNET: <https://disgenet.com/>

NCBI protein database: <https://www.ncbi.nlm.nih.gov/protein/>

SIFT: <https://sift.bii.a-star.edu.sg/>

PolyPhen-2: <http://genetics.bwh.harvard.edu/pph2/>

PredictSNP: <https://loschmidt.chemi.muni.cz/predictsnp/>

PhD-SNP: <https://snps.biofold.org/phd-snp/phd-snp.html>

PANTHER: <https://pantherdb.org/>

PROVEAN: <https://www.jcvi.org/research/provean>

Meta-SNP: <https://snps.biofold.org/meta-snp/>

SNAP2: <https://bioinformaticshome.com/db/tool/Snap2>

I-Mutant 2.0: <https://folding.biofold.org/cgi-bin/i-mutant2.0.cgi>

INPS-MD: <https://inpsmd.biocomp.unibo.it/>

iStable: <http://predictor.nchu.edu.tw/iStable/>

MUpro: <https://mupro.proteomics.ics.uci.edu/>

DynaMut2: <https://biosig.lab.uq.edu.au/dynamut2/>

HOPE: <https://www3.cmbi.umcn.nl/hope/input/>

MutPred2: <http://mutpred.mutdb.org/>

Swiss-PDB Viewer 4.1: <https://spdbv.unil.ch/>

Missense3D: <https://missense3d.bc.ic.ac.uk/>

InterPro: <https://www.ebi.ac.uk/interpro/>

STRING v.11.0: <https://string-db.org/>

Cytoscape: <https://cytoscape.org/>

SWISS-MODEL: <https://swissmodel.expasy.org/>

ProSA-web: <https://prosa.services.came.sbg.ac.at/prosa.php>

QMEAN: <https://swissmodel.expasy.org/qmean/>

ERRAT and Ramachandran plot analysis: <https://saves.mbi.ucla.edu/>

CASTpFold: <https://cfold.bme.uic.edu/castpfold/>

**Results**

**Q1]** Provide a rationale for merging SNPs (e.g., sequence redundancy, variant clustering, or updated annotations).

**Response:** Thank you for your thoughtful comment. We have now provided a rationale for merging certain SNPs in the revised manuscript (**page 13, line 358, 359**).

“During data curation, 5 variants were merged based on sequence redundancy, variant clustering, or updated annotations.”

**Q2]** Define and standardize terminology such as “deleterious,” “damaging,” or “high-risk” based on prediction thresholds or consensus across tools.

**Response:** Thank you for highlighting this important point. We defined and standardized terminology such as “deleterious,” “damaging,” or “high-risk” into our revised manuscript version.

The term “deleterious/damaging” specifically refers to SNPs predicted to significantly affect protein function, stability, or structure; potentially disease-associated. However, nsSNPs consistently predicted as deleterious or damaging across all eight tools were classified as common deleterious (**page 14, line 406-410**).

The term “high-risk” specifically refers to variants that significantly affect protein function, stability and structural destabilization across all computational prediction tools (e.g., I-MUTANT, MUpro, INPS-MD, iStable, DynaMut2, Missense3D, MutPred2, Swiss-PDB Viewer, and HOPE) suggesting a higher potential relevance to disease pathology (**page 20, line 545-548**).

**Q3]** Highlight whether the 10 predicted nsSNPs lie within functional domains of ApoE (e.g., receptor-binding or lipid-interaction regions).

**Response:** Thank you for your valuable suggestion. We highlighted whether the 10 predicted nsSNPs lie within functional domains into our revised manuscript version (**page 21, line 561-567**)

“Our analysis identified that 4 common deleterious nsSNPs (L107P, L122P, Y162C, and A208P) are located within functional domains of the ApoE protein. Additionally, 3 nsSNPs (R178W, R180H, and L185P) were situated within the hinge region, while the remaining 3 variants (W308G, P311H, and L323P) were located outside both functional domains and the hinge region those are shown in Fig. 5. Notably, the high-risk nsSNPs L107P and L122P were positioned within the receptor-binding domain.”

**Q4]** Briefly describe what Table 3 shows and how it supports the study’s conclusions.

**Response:** Thank you for your valuable suggestion. We have now provided a brief description of Table 3 to clarify its significance into our revised manuscript (**page 19, line 524-527**).

“Table 3 emphasized significant impacts on protein stability, and structural integrity across all employed computational prediction tools, including I-MUTANT, MUpro, INPS-MD, iStable, DynaMut2, Missense3D, MutPred2, Swiss-PDB Viewer, and HOPE. The consistent predictions across multiple computational platforms enhance the reliability of the findings.”

**Q5]** Ensure all tables and figures are cited sequentially and consistently (e.g., Table 3, S3 Table).

**Response:** Thank you for your careful observation. We have thoroughly reviewed the manuscript to ensure that all tables and figures are cited sequentially and consistently throughout the text. Any inconsistencies in numbering or referencing have been corrected to maintain clarity and proper alignment between the text and the corresponding data presentations.

**Q6]** If available, compare findings to previously published experimental or computational results.

**Response:** Thank you for your valuable suggestion. We have now incorporated a comparison of our findings with previously published computational study into our revised manuscript version (**page 34, line 949-951**).

**Q7]** Provide UniProt or PDB ID of the ApoE structure/template used for homology modeling in SWISS-MODEL.

**Response:** Thank you for your valuable feedback. In response, we have included the PDB ID (1B68) of the ApoE structure/template utilized for homology modeling in SWISS-MODEL in the revised version of our manuscript (**page 23, line 623**).

**Q8]** The L122P mutation shows higher Aβ binding affinity (ΔG = –6.6 kcal/mol). Discuss its functional implications, such as gain-of-function effects that may facilitate Aβ aggregation.

**Response:** Thank you for your insightful comment. We expanded the discussion to address the functional implications of the L122P mutation’s higher amyloid-beta (Aβ) binding affinity (ΔG = –6.6 kcal/mol) compared to wild-type (ΔG = –5.5 kcal/mol) into our revised manuscript version (**page 25, line 684-687**).

“This increased binding potential has functional implications: stronger ApoE (L122P)–Aβ interactions represent a gain-of-function effect that could promote Aβ aggregation through decrease Aβ clearance, and increase deposition in the brain exacerbating AD pathology.”

**Q9]** Clarify the genetic background of the modeled ApoE sequence, specifically, whether it corresponds to ApoE2, E3, or E4 isoforms. This is essential for interpreting pathogenic relevance.

**Response:** Thank you for your important observation. We have now clarified the genetic background of the modeled ApoE sequence in the revised manuscript (**page 23, line 624-628**).

“The modelled ApoE sequence corresponds to the ApoE4 isoform, which is defined by the presence of arginine at both positions 112 and 158, determined by the SNPs rs429358 and rs7412. This isoform is the most significant genetic risk factor for late-onset Alzheimer’s disease and is known to alter ApoE structure, lipid metabolism, and amyloid-beta (Aβ) interaction dynamics.”

**Discussion**

**Q1]** Explicitly state which ApoE isoform (E3 or E4) was modeled, as isoform context affects both structure and Aβ binding behavior.

**Response:** Thank you for your important observation. We have now explicitly stated the ApoE isoform used in our structural modeling (**page 32, line 885-889**).

“Structural modeling was conducted using the ApoE4 isoform, the most prevalent variant. ApoE4, characterized by arginine at positions 112 and 158, exhibits distinct domain interactions that promote amyloid aggregation and reduce clearance (13). Using this isoform allows for focused analysis while avoiding confounding effects from the ApoE4-specific domain–domain interaction.”

**Q2]** Justify the selection of L122P and L107P by indicating their position within functionally significant regions.

**Response:** Thank you for your valuable comment. We have now provided a clear justification for the selection of L122P and L107P mutations based on their positional relevance within functionally significant regions of the ApoE protein through domain identification by web-based computational tool, InterPro (**page 29, line 798-802**).

“Both mutations are located within the receptor-binding domain (residues 1–167) of ApoE, a region critical for interaction with low-density lipoprotein receptor-related proteins (e.g., LRP1 and LRP8) and the amyloid precursor protein (APP)—interactions essential for the regulation of amyloid-β (Aβ) transport and clearance.”

**Q3]** Provide biological context for Cytoscape network results, e.g., how ApoE mutations may affect interactions with APP or LRP1.

**Response:** Thank you for your valuable suggestion. We provided biological context for Cytoscape network results into our revised manuscript version (**page 31, line 865-878**).

“Cytoscape provides biological context by enabling visualization and analysis of molecular interaction networks, helping identify key genes, pathways, and functional modules in complex systems. Our Cytoscape analysis identified ApoE as a central hub protein, exhibiting strong interactions with multiple key proteins, including CLU, APOB, APP, APOC2, APOC1, APOA1, LRP1, LRP8, APOA2, and APOC3. Notably, ApoE showed substantial connectivity with Aβ-associated proteins such as APP, CLU, and LRP1, characterized by high betweenness and closeness centrality scores. These interactions are critical for signalling cascades, amyloid precursor protein (APP) processing, and neuroprotective functions (75,76). The receptor-binding domain of ApoE plays a pivotal role in mediating APP metabolism and Aβ clearance, primarily through interactions with LRP1 and LRP8, which facilitate neuronal lipid uptake and regulate amyloid burden. Structural alterations in ApoE’s receptor-binding domain, such as those resulting from the L122P or L107P variants, may disrupt its interaction network, potentially impairing Aβ homeostasis and diminishing neuroprotective capacity, thereby contributing to neurodegenerative disease mechanisms.”

**Q4]** Acknowledge the speculative nature of conclusions drawn from in silico predictions. Use cautious language (e.g., “predicted to impair” instead of “impairs”).

**Response:** Thank you for your thoughtful recommendation. We corrected the statement such as “predicted to impair” instead to “impair” in our revised manuscript version (**page 31, line 862**).

**Q5]** Explain how proline substitutions (e.g., at position 122) may disrupt local structural motifs, exposing hydrophobic regions that promote Aβ interaction and aggregation.

**Response:** Thank you for your valuable suggestion. We included the explanation of structural implications of proline substitutions, particularly the L122P mutation into our revised manuscript version (**page 30, line 845-850**)

“Unlike leucine, proline has a rigid cyclic structure that introduces kinks into α-helices, disrupts backbone hydrogen bonding, and destabilizes local secondary structures. These structural distortions expose normally buried hydrophobic residues, promoting non-specific interactions and enhancing Aβ binding. Such mutations significantly impact protein stability, often leading to denaturation and loss of function, thereby contributing to disease progression.”

**Q6]** Include a brief comparison with existing literature on rare or emerging ApoE variants (e.g., R176C, C130R) to validate findings or identify novel trends.

**Response:** Thank you for your valuable suggestion. We have now incorporated a brief comparison with existing literature on rare or emerging ApoE variants, such as R176C and C130R, to contextualize our findings into discussion section of our revised manuscript version (**page 34, line 949-954**).

“Variants such as R176C and C130R, located within the receptor-binding domain, have been shown to disrupt lipidation and receptor interactions, thereby contributing to AD-related pathology (83,84). Similarly, our identification of the L122P high-risk variant, also within this domain, with increased Aβ binding affinity, suggests its potential role in modulating ApoE function and contributing to AD pathology.”

**Conclusion**

**Q1]** Emphasise novelty: state whether L122P and L107P are newly reported or previously uncharacterized high-risk variants.

**Response:** Thank you for your valuable comment. We have now emphasized the novelty of our findings by clearly stating that the L122P and L107P mutations are previously uncharacterized high-risk variants into the conclusion section of our revised manuscript version (**page 35, line 989-992**).

“this study reports newly identified and previously uncharacterized high-risk ApoE variant, L122P, contributing to altered Aβ binding and structural destabilization, and offers insights for precision-medicine approaches targeting variant-specific mechanisms in Alzheimer’s disease.”

**Q2]** Mention potential clinical relevance, use of findings for diagnostic markers or therapeutic targets.

**Response:** Thank you for your valuable suggestion. We have now addressed the potential clinical relevance of our findings in the revised manuscript (**page 35, line 982-984**).

“This variant could potentially serve as novel diagnostic biomarkers for early identification of individuals at increased risk for AD, particularly if validated in future genetic association studies.”

**Q3]** Highlight any novel binding mechanisms or interactions uncovered during docking.

**Response:** Thank you for your insightful suggestion. We have now highlighted novel binding mechanisms and interactions observed during the docking analysis (**page 35, line 977-981**).

“Molecular docking and dynamics simulations revealed that the leucine-to-proline substitution at position 122 introduces a novel binding interface, likely due to the rigidity of proline’s cyclic structure, which disrupts local secondary motifs and exposes hydrophobic surfaces that favor Aβ interaction. This altered binding impairs Aβ clearance, a core pathological mechanism in AD.”

**Q4]** Begin with a clear, impactful summary of the core discovery.

Suggested: "This study presents the first comprehensive in silico characterization of L122P and L107P ApoE variants, revealing their significant structural and binding alterations with Aβ, and their possible contribution to Alzheimer’s disease progression."

**Response:** Thank you for your helpful suggestion. We have now revised the opening of the manuscript to begin with a clear and impactful summary of the core discovery (**page 34, line 970-972**).

"This study presents the first comprehensive in silico characterization of L122P and L107P ApoE variants, revealing their significant structural and binding alterations with Aβ, and their possible contribution to Alzheimer’s disease progression."

**Q5]** Minimize vague expressions like “may impair” unless essential. Instead, use confident but appropriate language supported by predictions (e.g., “is predicted to impair Aβ clearance”).

**Response:** Thank you for your helpful recommendation. We have carefully revised the manuscript to minimize vague expressions such as “may impair” into the conclusion section (**page 35, line 981**).

**Reviewer 03#**

**Major Comments**

**Q1]** The current title is overly generic. It should be rephrased to better reflect the study’s central hypothesis and mechanistic approach. Similarly, the keywords require revision to include relevant technical terms that capture the essence of the work.

**Response:** Thank you for your valuable feedback regarding the title and keywords. According to your suggestion, we rephrased our title as well as revised our keywords.

Title: “Computational Prediction of High-Risk Non-Synonymous SNPs in Human ApoE and Their Structural Impact on Amyloid-β Interaction in Alzheimer’s Disease Pathogenesis.” (**page 1, line 1, 2**).

Keyword: “Alzheimer’s disease, Apolipoprotein E (ApoE), non-synonymous SNPs, Receptor-binding domain, High-risk nsSNPs, Amyloid-β (Aβ)-ApoE interaction.” (**page 2, line 63, 64**).

**Q2]** The abstract suffers from grammatical issues and poorly structured sentences. It is heavily loaded with results but lacks coherence and a logical flow. Importantly, there is no concluding remark or forward-looking statement. The parenthetical phrase "excluding five" is unclear and unnecessary—please clarify its relevance or remove it.

**Response:** Thank you for your detailed and constructive feedback on the abstract. In response, we have addressed the grammatical errors and restructured poorly constructed sentences. We also addressed the issue of excessive result reporting by improving coherence and ensuring a more logical flow in the specified section (**pages 1–2, lines 20–61**). We added concluding remarks and forward-looking statement (page 2, line 58-61) and lastly, we removed the phase “excluding five”.

**Q3]** The introduction presents a very basic overview and fails to build a strong scientific context. It should be rewritten to include the genetic and molecular mechanisms underlying ApoE’s role in AD. A compelling introduction must engage the reader, present relevant background, and clearly articulate the study’s rationale. Additionally, recent statistics and literature that support the gene-disease connection should be incorporated to establish relevance.

**Response:** Thank you for your valuable and detailed feedback. In response, we revised the introduction to incorporate the genetic and molecular mechanisms underlying ApoE’s role in Alzheimer’s disease (**page 3, lines 79–82**). We also integrated recent literature and key findings from genome-wide association studies (GWAS), epidemiological data, and experimental research that robustly support the gene-disease association (**page 3, lines 70–72, 87–88; page 4, lines 103–106**). Furthermore, the study’s rationale has been clearly articulated in **page 5, lines 141–142 and 151–153**.

**Q4]** Methodology: This section requires significant enhancement. The SNP retrieval process should be validated against other reputable databases. Clarify what is meant by "common deleterious nsSNPs in ApoE." The heading of Table 1 should be revised for clarity, and the inclusion of input parameters in the table must be justified. Also, the rationale for using SWISS-MODEL for homology modelling should be clearly explained. Why was this tool chosen over more robust alternatives?.

**Response:** Thank you for your valuable and detailed feedback regarding the methodology. In response, we made several revisions to enhance the clarity, transparency, and scientific rigor of the section. The SNP retrieval pipeline has been expanded to incorporate cross-validation using multiple reputable databases, including DisGeNET and ClinVar (**page 6, lines 167–177**), ensuring the reliability of the variant data. The term "common deleterious nsSNPs" has been clarified to avoid ambiguity and improve interpretability (**pages 8–9, lines 231–232**). Additionally, the heading of Table 1 has been revised for greater clarity and alignment with the table content (**page 7, lines 219–220**). We have also provided a detailed rationale for selecting SWISS-MODEL as the tool for homology modeling, emphasizing its accuracy, reliability, and suitability for modeling ApoE structures (**pages 10–11, lines 291–297**).

**Q5]** Results: The results section lacks depth and needs a more detailed discussion. In Table 3, the footnote should be expanded to explain the data presented. Elaborate on domain identification in ApoE and the implications of locating high-risk nsSNPs within these domains. The biological relevance of the protein-protein interaction findings must be thoroughly discussed. The linkage between different outputs in Table 4 needs clarification. Justify the inclusion of molecular docking and explain the impact of the L122P mutation—especially the claim regarding active site changes. In Table 5, 2D interactions should be labelled clearly, including the nature of bonding (e.g., hydrogen bonding, hydrophobic interactions).

**Response:** We totally agree with your opinion. As per your recommendation expanded the footnote in Table 3 to provide a clearer explanation of the data presented (**page 17, lines 456–462**). Elaborated on domain identification in ApoE and the implications of locating high-risk nsSNPs within these domains (**page 21, line 558-567**). Furthermore, we have discussed the biological relevance of the protein–protein interaction findings to better contextualize their role in disease pathogenesis (**pages 21–22, lines 586–594**). The relationship among the different outputs presented in Table 4 has been clarified to enhance interpretability (**page 23, lines 637–641**). Additionally, we have justified the inclusion of molecular docking analysis and provided a detailed explanation of the L122P mutation's impact, particularly in relation to changes observed in the active site (**page 25, lines 677–687**). Lastly, we have clearly labeled the 2D interaction diagrams in Table 5, specifying the types of molecular interactions, such as hydrogen bonding and hydrophobic contacts (**pages 25–26, line 701**).

**Q6]** Discussion: This section largely reiterates the results without offering critical analysis. The discussion should interpret findings in light of existing literature and proposed mechanisms. Potential limitations of the study should be acknowledged. Emphasize that the findings demonstrate associations and do not establish causality. Broaden the discussion to include how these results fit into larger biological or disease models.

**Response:** Thank you for your insightful feedback regarding the discussion section. In response, we thoroughly revised the section to enhance its depth and clarity and contextual relevance of the discussion. A more critical analysis of the findings has been provided, including an interpretation of the identified nsSNPs in relation to existing literature (**page 34, lines 948–951**). The limitations of the study have been clearly acknowledged (**pages 33–34, lines 940–947**), and it has been emphasized that the findings should be interpreted as associative rather than causative (**page 34, lines 958–960**). Additionally, the discussion has been expanded to explore how these results align with broader biological mechanisms and disease models, offering greater insight into their relevance within the context of Alzheimer’s disease (**page 34, lines 952–957**).

**Q7]** The conclusion is repetitive and lacks impact. Provide a concise summary of the major findings, followed by a forward-looking statement suggesting future directions or potential applications of the research.

**Response:** Thank you for your valuable feedback regarding the conclusion section. In response, we addressed the issues of repetition and lack of impact by streamlining the content to provide a concise summary of the key findings (**pages 34–35, lines 970–991**). Additionally, we have included a forward-looking statement outlining potential future research directions and applications of the study’s outcomes (**page 35 line 981-983, 988-991**).

**Q8]** References: Many references are outdated. Replace or supplement these with more recent studies published within the last five years to ensure the literature review is current and relevant.

**Response:** Thank you for your valuable observation regarding the reference list. In response, we conducted a comprehensive review of recent literature and have updated the manuscript to include the most relevant and up-to-date studies published within the last 15-20 years.

Minor Comments

**Q9]** Language and Grammar: The manuscript requires thorough proofreading and professional language editing. Attention should be given to sentence structure, grammar, and consistency in scientific terminology to enhance readability.

**Response:** Thank you for your valuable feedback regarding the language and grammar of the manuscript. In response, we carefully revised the entire manuscript, addressing grammatical issues, sentence structure, and terminological consistency.

**Q10]** Figures and Schematic Diagrams: The workflow diagram appears basic and visually unappealing. Consider redesigning it using graphical elements that enhance comprehension and aesthetic quality. For inspiration, refer to workflows presented in the following publications:

DOI: 10.1021/acsomega.2c04871

DOI: 10.1021/acsptsci.2c00212

DOI: 10.1021/acs.jcim.0c00488.

**Response:** Thank you for your valuable suggestion regarding the quality of the workflow diagram. In response, we redesigned the workflow diagram using advanced graphical tools to improve its clarity, structure, and overall aesthetic quality.

**Q11]** Figures and Visual Quality: Ensure all figures are of high resolution and stylistically consistent. Figure legends should be self-explanatory, clearly describing all elements shown, and aiding interpretation without requiring reference to the main text.

**Response:** Thank you for your valuable feedback regarding the figures and their visual quality. In response, we carefully updated all figures to high-resolution versions to ensure clarity and visual sharpness. Additionally, we have revised the figure designs to maintain stylistic consistency across the manuscript, including uniform color schemes, font sizes, and layout structures.
